# Complication rates of 16- and 18-gauge needles for native kidney biopsies: a systematic review and proportional meta-analysis

**DOI:** 10.1080/0886022X.2026.2665032

**Published:** 2026-05-25

**Authors:** Marie Møller, Tue H. Andersen, Rikke Borg, Robin Christensen, Iain Bressendorff, Emilie K. Hansen, Rasmus Rønnemoes, Signe K. Jakobsen, Karina Haar, Frederik Persson, Peter Rossing, Ditte Hansen

**Affiliations:** a Department of Nephrology, Copenhagen University Hospital – Herlev and Gentofte, Herlev, Denmark; b Danish Diabetes Knowledge Center, Department of Education, Copenhagen University Hospital – Steno Diabetes Center Copenhagen, Herlev, Denmark; c Department of Medicine, Zealand University Hospital, Roskilde, Denmark; d Department of Clinical Medicine, University of Copenhagen, Copenhagen, Denmark; e Section for Biostatistics and Evidence-Based Research, the Parker Institute, Bispebjerg and Frederiksberg Hospital, Copenhagen, Denmark; f Research Unit of Rheumatology, Department of Clinical Research, University of Southern Denmark, Odense University Hospital, Odense, Denmark; g Department of Complications Research, Copenhagen University Hospital – Steno Diabetes Center Copenhagen, Herlev, Denmark

**Keywords:** Biopsy needle size, complications, kidney biopsy, meta-analysis, systematic review, major bleeding complications

## Abstract

This systematic review and meta-analysis evaluated complication rates and diagnostic yield reported in studies of adult native kidney biopsy using 16-gauge (16 G) or 18-gauge (18 G) needles. We included randomized trials and cohort studies of real-time ultrasound-guided biopsies, including case series. MEDLINE, Embase, and CENTRAL were searched through October 2024. Two reviewers independently performed study selection, data extraction, and risk of bias assessment using Joanna Briggs Institute tools. Random-effects meta-analyses estimated pooled proportions with 95% confidence intervals (CI), and univariable random-effects meta-regression explored study-level associations with major complications, transfusion, or gross hematuria. We screened 4,499 titles and abstracts and reviewed 319 full-text articles; 62 studies comprising 68 biopsy series were included. The pooled major complication rate in studies using 16 G needles was 1.83% (95% CI: 1.20–2.79) and 1.29% (95% CI: 0.78–2.13) in studies using 18 G needles, with no statistically significant difference. Mean glomerular yield was 18.8 with 16 G and 17.5 with 18 G needles. In study-level meta-regression, studies with higher prevalence of acute kidney injury, lower mean estimated glomerular filtration rate, or lower mean hemoglobin reported higher pooled complication rates. Most studies were single-arm cohorts; between-needle differences therefore reflect indirect study-level contrasts. Interpretation is limited by retrospective design and heterogeneity across studies. Overall, studies using both needle sizes reported low complication rates and similar diagnostic yield, although definitions and reporting varied. Direct comparative studies are needed to determine whether meaningful differences exist.

## KEY LEARNING POINTS

What was known

- Kidney biopsy is essential for diagnosing many medical kidney diseases and guiding treatment, requiring adequate tissue samples for subsequent analyses.
- Modern biopsy techniques use spring-loaded, ultrasound-guided devices with smaller gauge needles, but optimal needle size remains uncertain.
- While 14G needles are associated with higher complication risk, evidence on whether 16G or 18G needles differ in safety or diagnostic yield is conflicting.

This study adds

- In this systematic review and meta-analysis of 62 studies involving 22,208 adult native kidney biopsies, we found no statistically significant difference in major complication rates between studies using 16G and 18G needles.
- In study-level meta-regression analyses, studies with a higher prevalence of acute kidney injury, lower mean estimated glomerular filtration rate, and lower mean hemoglobin reported higher pooled major complication rates.

Potential impact

- Studies using both 16G and 18G needles reported similarly low major complication rates, although comparisons are based on indirect evidence with substantial heterogeneity.
- Findings support the ethical feasibility and clinical relevance of a large, randomized controlled trial to definitively guide needle size selection in native kidney biopsies.

## Introduction

A kidney biopsy is an essential tool in the diagnostic workup of kidney diseases. The kidney biopsy was introduced for routine use in the 1950s [1] and has provided clinicians with great insight into the pathology of kidney diseases. Many medical kidney diseases cannot be diagnosed without sufficient tissue for evaluation by light microscopy, immunofluorescence, and electron microscopy. Because morphological findings from kidney biopsies substantially influence treatment decisions in a large proportion of patients [1–3], obtaining adequate samples is essential.

The current standard procedure for kidney biopsy in most centers involves using real-time ultrasonographic guidance and an automated spring-loaded biopsy device [2–7]. These advances have replaced traditional large-bore manual needles with smaller-bore automatic needles with improved sampling and safety profiles [8]. Several different needle sizes are available for kidney biopsy, but the optimal needle size has not been established. The kidney biopsy procedure is associated with a small risk of severe bleeding complications such as gross hematuria, blood transfusion, or surgery/arterial embolization. Predictors of complications associated with the procedure vary across studies, but it is well established that the risk increases with a needle of 14-gauge (14 G) or larger [3,9–11]. The literature disagrees on whether the risk differs between the most used needle sizes of 16-gauge (16 G) and 18-gauge (18 G).

The biopsy equipment and technique must balance the need for adequate tissue samples against the risk of procedural complications, and therefore the aim of this systematic review and meta-analysis was to compare complication rates and biopsy adequacy reported in studies using 16 G versus 18 G needles for adult native kidney biopsy. The primary objective was to compare the risk of major complications between 18 G and 16 G needles in adult native kidney biopsy. Our secondary objectives were to describe the average number of glomeruli harvested with the two needle sizes, the number of inconclusive biopsies, and to explore other risk factors for complications available in the eligible literature.

## Methods

### Protocol and registration

The review protocol was registered in PROSPERO (CRD42024511185), and reporting followed PRISMA guidelines [12,13] (Table S1).

### Eligibility criteria

Studies were eligible if they represented arms from randomized trials and prospective or retrospective cohort studies involving adults (≥18 years) with medical kidney disease who underwent real-time ultrasound-guided native kidney biopsy using a 16 G or 18 G needle. Studies using one or multiple needle sizes were eligible if data could be disaggregated by needle size. Major complication data had to be available, defined as transfusion, embolization, nephrectomy, surgery, or death. No publication year restrictions applied.

### Information sources and search strategy

We searched Medline, Embase, and CENTRAL 3rd March 2023 and updated the search 11th October 2024 using a strategy combining MeSH terms (e.g. *Biopsy, Needle/*, *Hemorrhage/*) and free-text terms (e.g. kidney$, nephro$, renal). An information specialist (THA), with formal training in systematic review methodology and evidence-based healthcare, developed the search in Ovid MEDLINE, which was then adapted to Embase and CENTRAL. The strategy was tested against 22 key articles and peer reviewed by a second information specialist. Full details are available in the supplementary material (Supplementary Tables S2–S4). After study selection, references of included studies were identified using the software tool CitationChaser [14] and screened to find studies not retrieved in the database search.

### Study selection, data collection, and risk of bias assessment

We uploaded all search results to the web-based screening tool EPPI Reviewer 6 to streamline screening efficiency [15]. Duplicates were removed before initiating the study selection. The review proceeded in two steps: First, titles and abstracts were randomly assessed by two independent reviewers (MM, DH, FP, RB, KH, RRI). Exclusion criteria included: fewer than 10 biopsies, no native kidney biopsies, lack of real-time ultrasound guidance, no complication data, focus on kidney tumor biopsies, open or non-kidney biopsies, or publication type (review, editorial, case report). Disagreements were resolved by a third reviewer.

In step two, full texts of the remaining papers were assessed by two reviewers (MM, RRI, EKH, SKJ) using the same exclusion criteria. Additional exclusions were applied to studies lacking information on needle size or where data from mixed cohorts (e.g. including pediatric or transplant biopsies) could not be separated. Authors were contacted when relevant data were missing. Studies were excluded if key information could not be obtained. For data extraction, each included paper was assigned to one reviewer for data entry, followed by verification from a second reviewer (MM, EKH, SKJ, TMM). Disagreements were resolved through discussion and consensus. Articles in languages other than English, German, Danish, Swedish, or Norwegian were excluded due to language barriers, although they appeared eligible based on their titles and abstracts. These are listed in Supplementary Table S5.

We assessed risk of bias using the Joanna Briggs Institute (JBI) critical appraisal tools for cohort studies and RCTs, with scoring according to the recommendation by JBI guidelines (see Supplementary Material Figures S1 and S2) [16–18].

**Figure 1.**
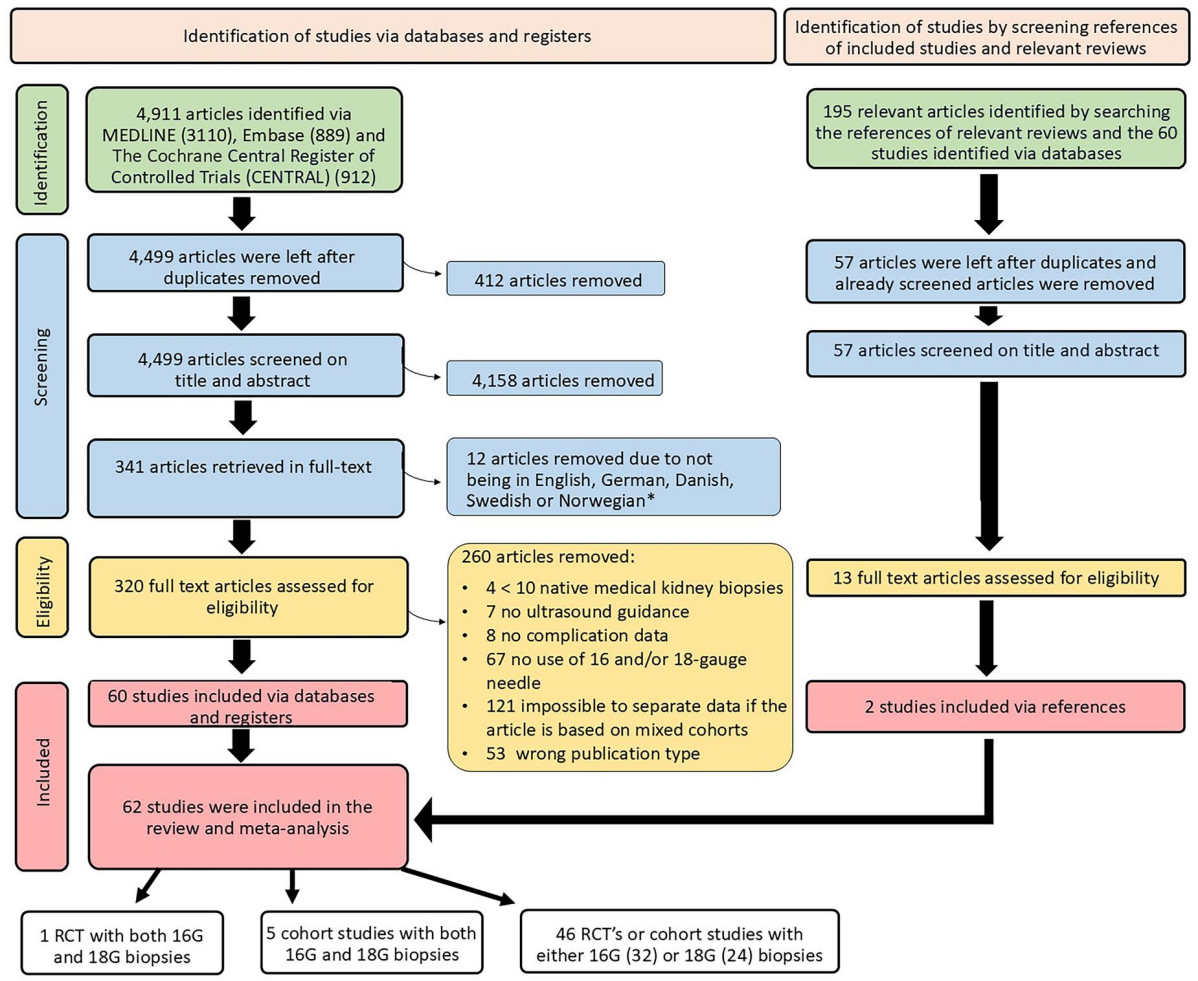
Preferred reporting items for systematic reviews and meta-analyses (PRISMA) flow chart. *Due to language barriers, we could not assess these articles fully. Thus, they are excluded from the synthesis but included in the supplementary data for others to read and analyze. G, gauge. RCT, Randomized controlled trial.

**Figure 2.**
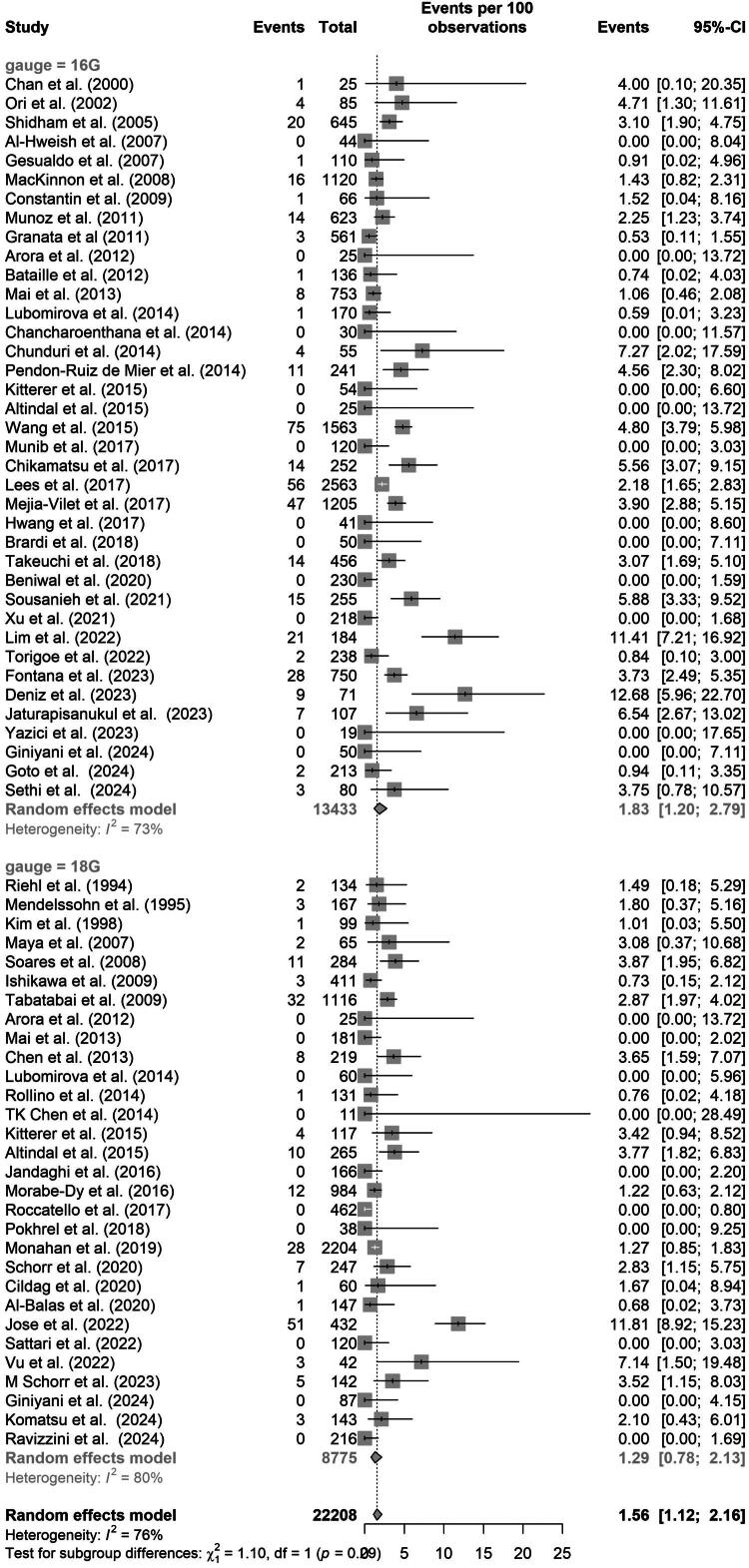
Proportion of major complications stratified by needle size. Random effects model testing difference between the two needle sizes and Major bleeding complications. CI, Confidence Interval. I^2^, Heterogeneity.

### Statistical methods

A proportional meta-analysis is a type of single-arm (or single-group) meta-analysis that pools proportions across studies to provide a summary estimate of the frequency of an event or characteristic [19]. That is, each study contributes a proportion, defined as the number of cases divided by the total sample size. A proportional meta-analysis is a type of single-arm meta-analysis that pools proportions across studies to estimate the frequency of an event or characteristic. For the evidence synthesis, each study contributed the proportion of major bleeding events, defined as the number of events divided by the total sample size. These study-specific proportions were synthesized to produce pooled estimates with corresponding 95% confidence intervals. A meta-analysis of proportions was conducted using a random-effects model with a random intercept logistic regression [20] and the maximum-likelihood estimator [21]. Log transformation was applied to approximate normal distribution and reduce bias in standard errors, followed by back-transformation to obtain the overall estimated proportion [22]. For studies enabling direct comparison of 16 G and 18 G needles, risk ratios (RR) were calculated, and these estimates were combined using a random-effects model with inverse variance weighting and the Hartung-Knapp adjustment [23].

Heterogeneity was assessed and interpreted based on the observed inconsistency using the I^2^ statistic, with thresholds of 25%, 50%, and 75% indicating low, moderate, and high heterogeneity [24]. Estimates with 95% confidence intervals are presented in a forest plot. We conducted univariable random-effects meta-regression to examine sources of heterogeneity [25], assessing associations between complication risk and age, sex, creatinine, hemoglobin, eGFR, blood pressure, sample size, biopsy indication (acute kidney injury or nephrotic syndrome), number of passes, and operator type (nephrologist vs radiologist).

Risk of bias across studies was assessed to determine whether methodological shortcomings affected reported major complication rates. Sensitivity analyses evaluated whether findings were robust or driven by higher-risk studies. Funnel plots assessed potential bias and small-study effects, plotting study proportions against standard errors, with larger, more precise studies toward the top and smaller, less precise studies spreading wider at the bottom. Contour-enhanced funnel plots (*p* ≤ 0.05) aided interpretation [24]. Analyses were performed using R 4.1.0 (2021-05-18) [26] with the packages metafor [27], meta [28], and ggplot2 [29].

## Results

We identified 4 911 articles through database search; after removing 412 duplicates, 4 499 were screened by title/abstract and language, and 319 underwent full-text review. An additional 195 articles were found *via* reference and review screening. In total, 62 studies were included in the final review and meta-analysis. Six reported on both needle sizes [30–35], 32 on 16 G only [10,11,36–65], and 24 on 18 G only [8,66–88] (Figure 1).

The total number of biopsies included in this review was 22 208, with 13 433 performed using a 16 G needle and 8 775 using an 18 G needle. The median number of biopsies per study was 145 (IQR 64–270), ranging from 11 to 2 563. Among the included studies, 9 were randomized controlled trials, 8 prospective cohorts, 42 retrospective cohorts, and 3 had a mixed design. The mean patient age ranged from 24 to 79 years (median 48), and the proportion of female participants ranged from 30% to 100% (median 45%). Twenty-two studies reported pre-biopsy systolic blood pressure, 38 reported creatinine levels, and 39 reported hemoglobin. Biopsy indication was available in 24 studies: 18 reported rates of acute kidney injury (AKI) and 21 of nephrotic syndrome. Nephrologists performed the procedure in 38 studies and automated spring-loaded needles were used in 47 studies. Data on gross hematuria were reported in 40 studies, and hematoma rates in 43. Routine post-biopsy ultrasound was performed in 37 studies (Table 1 and Supplementary Table S6). Risk of bias was rated high in 6 studies, moderate in 38, and low in 18 (Supplementary Figures S1 and S2). Major post-biopsy complications—defined as transfusion, embolization, nephrectomy, surgical intervention, or death—were consistently reported across included studies, whereas minor complications, such as asymptomatic hematomas or microscopic hematuria, were variably reported, particularly in retrospective studies.

**Table 1.** Characteristics of included studies.

Author (Country code)	Design	Inclusion period	Biopsies in the study (n)	Biopsies relevant for this review (n)	Mean age	Female (%)
**Studies with usage of both 16 G and 18 G needles.**						
Arora et al. (IN) [30]	RCT	2007–2008	50	16G: 25 18 G: 25	NA NA	NA NA
Lubomirova et al. (BG) [31]	R Cohort	2009–2013	230	16G: 170 18 G: 60	45.5** 45.5**	48.3** 48.3**
Kitterer et al. (DE) [32]	R Cohort	2008–2012	205	16G: 54 18 G: 117	58** 58**	39.0** 39.0**
Mai et al. (AU) [33]	R Cohort	2001–2010	934	16G: 753 18 G: 181	51.0 53.0	47.0 45.0
Altindal et al. (TR) [34]	R Cohort	2000–2013	290	16G: 25 18 G: 265	39.9** 39.9**	39.3** 39.3**
Giniyani et al. (US) [35]	R Cohort	2015–2023	137	16G: 50 18 G: 87	44.0 48.0	NA NA
**Studies with 16 G needles**						
Brardi et al. (IT) [36]	R Cohort	2012–2017	50	50	52.7	36.0
Chancharoenthana et al. (TH) [37]	RCT	2014***	30	30	53.6	60.0
Chunduri et al. (US) [10]	P Cohort	2010–2013	137	55	48.0	65.0
Shidham et al. (US) [38]	R Cohort	1981–2001	645	645	42.0	50.0
Bataille et al. (FR) [39]	R Cohort	2006–2010	943	136	52.2	36.0
Lim et al. (SG) [40]	R Cohort	2011–2015	184	184	54.1	44.0
Wang et al. (CN) [41]	R + P Cohort	2010–2012	1985	1563	40.0	39.5
Beniwal et al. (IN) [42]	R Cohort	2012–2017	230	230	64.0	29.6
MacKinnon et al. (GB-SCT) [43]	R Cohort	2000–2007	1120	1120	56.0	40.1
Takeuchi et al. (JP) [44]	R Cohort	2013–2017	456	456	65.7	37.7
Al-Hweish et al. (SA) [45]	RCT	2004–2006	44	44	NA	48.0
Munib et al. (PK) [46]	R Cohort	2013–2015	120	120	28.2	31.6
Munoz et al. (MX) [47]	R Cohort	1998–2008	623	623	34.4	70.5
Sousanieh et al. (US) [48]	P Cohort	2002–2019	592	255	46.0	42.0
Gesualdo et al. (IT) [49]	RCT + Cohort	2005–2007	110	110	45.7	NA
Constantin et al. (CA) [11]	R Cohort	2005–2007	121	66	54.6	50.0
Xu et al. (CN) [50]	P Cohort	2016–2017	218	218	45.7	52.3
Pendon-Ruiz de Mier et al. (ES) [51]	P Cohort	2009–2013	241	241	49.0	44.0
Chikamatsu et al. (J P) [52]	R Cohort	2013–2016	252	252	62.0	39.0
Lees et al. (GB-SCT) [53]	R Cohort	2000–2014	2563	2563	57.0	42.6
Fontana et al. (IT) [54]	R Cohort	2010–2020	750	750	52.2	41.2
Granata et al. (IT) [55]	R Cohort	1995–2009	561	561	45.9	43.0
Torigoe et al. (JP) [56]	R Cohort	2017–2020	238	238	54.0	46.6
Mejia-Vilet et al. (MX) [57]	R + P Cohort	2008–2016	1205	1205	33.0	68.8
Hwang et al. (KR) [58]	R Cohort	2014–2015	41	41	40.7	36.6
Chan et al. (CA) [59]	P Cohort	1998–1999	25	25	NA	NA
Ori et al. (IL) [60]	P Cohort	1995	94	85	52.9	47.1
Deniz et al. (TR) [61]	P Cohort	2020–2022	71	71	47.9	53.5
Jaturapisanukul et al. (TH) [62]	RCT	2017–2019	107	107	41.6	66.0
Yazici et al. (TR) [63]	R Cohort	2001–2022	19	19	27.4	100
Goto et al. (JP) [64]	R Cohort	2018–2023	213	213	56.0	48.4
Sethi et al. (IN) [65]	RCT	2021–2022	80	80	44.1	37.5
Studies with 18 G needles						
Kim et al. (KR) [66]	RCT	1994–1997	166	99	35.7	46.5
Jandaghi et al. (IR) [67]	RCT	2015–2016	166	166	43.0	46.4
Soares et al. (US) [68]	R Cohort	1996–2006	289	284	57.4	43.6
Morabe-Dy et al. (PH) [69]	R Cohort	2012–2015	984	984	38.3	56.2
Jose et al. (IN) [70]	R Cohort	2014–2018	432	432	39.1	36.3
Sattari et al. (IR) [71]	RCT	2017–2020	120	120	45.3	51.7
Schorr et al. (CA) [72]	R Cohort	2012–2017	617	247	57.0*	36.5*
Ishikawa et al. (JP) [73]	R Cohort	2001–2006	411	411	45.2	44.5
Mendelssohn et al. (Ca) [74]	P Cohort	1992–1994	544	167	NA	NA
Roccatello et al. (IT) [75]	P Cohort	2000–2016	462	462	54.7	39.0
Pokhrel et al. (NP) [76]	RCT	2016	76	38	33.9	37.0
Maya et al. (US) [77]	R Cohort	2004–2005	129	65	43.0	61.0
Cildag et al. (TR) [78]	R Cohort	2017–2018	60	60	49.5	36.7
Tabatabai et al. (US) [79]	R Cohort	1995–2007	1116	1116	45.1	56.3
Chen et al. (US) [80]	R Cohort	1993–2007	219	219	36.5	85.6
Rollino et al. (IT) [81]	R Cohort	1974–2012	131	131	78.7	45.0
TK Chen et al. (US) [82]	R Cohort	2001–2012	11	11	24.0	100
Monahan et al. (US) [83]	R Cohort	2005–2015	2204	2204	59.0	42.1
Vu et al. (US) [84]	R Cohort	2006–2021	42	42	52.9	45.2
Al-Balas et al. (US) [85]	R Cohort	2017–2019	147	147	48.0	49.0
M Schorr et al. (CA) [86]	R Cohort	2018–2023	400	142	56.0	40.2
Komatsu et al. (JP) [87]	R Cohort	2016–2022	224	143	64.0	48.3
Ravizzini et al. (BR) [88]	R Cohort	2008–2021	445	216	40.2	NA
Riehl et al. (DE) [8]	R Cohort	1990–1993	458	134	45.9	43.0

* Before the biopsy.

** Data only available for the whole cohort.

*** Study period not mentioned, and the presented year is time of publication.

Country codes: AU, Australia. BG, Bulgaria. BR, Brazil. CA, Canada. CN, China. DE, Germany. ES, Spain. FR, France. GB-SCT, Scotland. IL, Israel. IN, India. IR, Iran. IT, Italy. JP, Japan. KR, South Korea. MX, Mexico. NP, Nepal. PH, Philippines. PK, Pakistan. SA, Saudi Arabia. SG, Singapore. TH, Thailand. TR, Turkey. US, United States. Cr, Creatinine. G, Gauge. Hgb, hemoglobin. NA, Not Applicable. Nephro., Dept. of Nephrology. P, prospective. R, retrospective. Radio., Dept. of Radiology. RCT, Randomized controlled trial.

For other covariates (blood pressure, mean creatinine, mean hemoglobin, department performing the biopsy, and type of biopsy needle) see Supplementary Table S6.

### Major complication

As illustrated in Figure 2, the overall major complication rate was 1.83% (95% CI, 1.20% to 2.79%) for 16-gauge needles and 1.29% (95% CI, 0.78% to 2.13%) for 18-gauge needles. Comparison of these pooled proportions, treating the 16 G and 18 G study groups as independent, showed no statistically significant difference in complication rates by needle size: estimate difference 0.54%; 95% CI: −0.74% to 1.82%. Heterogeneity was moderate in the 16 G group (I^2^ = 73%) and high in the 18 G group (I^2^ = 80%). In the analysis restricted to 18 high-quality studies (4,003 16 G biopsies and 2,743 18 G biopsies), complication rates increased to 2.69% (95% CI: 1.38% to 5.20%) for 16 G and 1.36% (95% CI: 0.58% to 3.18%) for 18 G, with a statistically significant difference (estimate difference 1.33%; 95% CI: 0.8% to 2.02%). Heterogeneity remained high for 16 G (I^2^ = 83%) and was low for 18 G (I^2^ = 4.1%) (Figure S3). This observed difference should be interpreted cautiously, as it may reflect differences in patient populations, center practices, or reporting rather than a causal effect of needle size. In a meta-analysis of the six studies with direct comparison, we again found no difference in major bleeding risk between the two needle sizes (Figure S4 in Supplementary).

In the univariate meta-regression, major complications were significantly associated with AKI, eGFR, and hemoglobin (Table S7). These findings suggest that AKI, low eGFR, and low hemoglobin may be predictors of major complications. No other variables showed significant associations. A t-test comparing 16 G and 18 G biopsies showed no significant difference in any of the three variables: eGFR 9.13 (95% CI: −14.35 to 32.61), AKI −0.16% (–18.02 to 17.70), and hemoglobin 0.44 (–0.12 to 1.00). When assessed individually, transfusion, embolization, and death showed no significant differences by needle size (Table 2; Figures S5–S7 in Supplementary).

**Table 2.** Summary of proportions of major bleeding complications.

jlfw	Overall proportion (%) with 95% CI	Proportion for 18G needles (%) with 95% CI	Proportion for 16G needles (%) with 95% CI	Estimate difference with 95% CI
Major complications*	1.56 (1.12 to 2.16)	1.29 (0.78 to 2.13)	1.83 (1.20 to 2.79)	0.54 (−0.74 to 1.82)
Transfusion	1.34 (0.96 to 1.86)	1.15 (0.73 to 1.81)	1.53 (0.98 to 2.40)	0.38 (−0.71 to 1.47)
Embolization	0.30 (0.19 to 0.47)	0.28 (0.12 to 0.62)	0.33 (0.21 to 0.53)	0.05 (−0.34 to 0.44)
Death	0.01(0.00 to 0.04)	0.01 (0.00 to 1.49)	0.02 (0.00 to 0.06)	0.01 (−1.47 to 1.49)

* Major complications defined as transfusion, embolization, nephrectomy, surgical intervention, and/or death.

CI, confidence interval. G, Gauge.

In the univariate meta-regression for transfusion risk, eGFR and hemoglobin showed the same associations. Additionally, high creatinine and systolic blood pressure were associated with increased risk (Table S8 Supplementary).

### Publication bias

**Figure 3.**
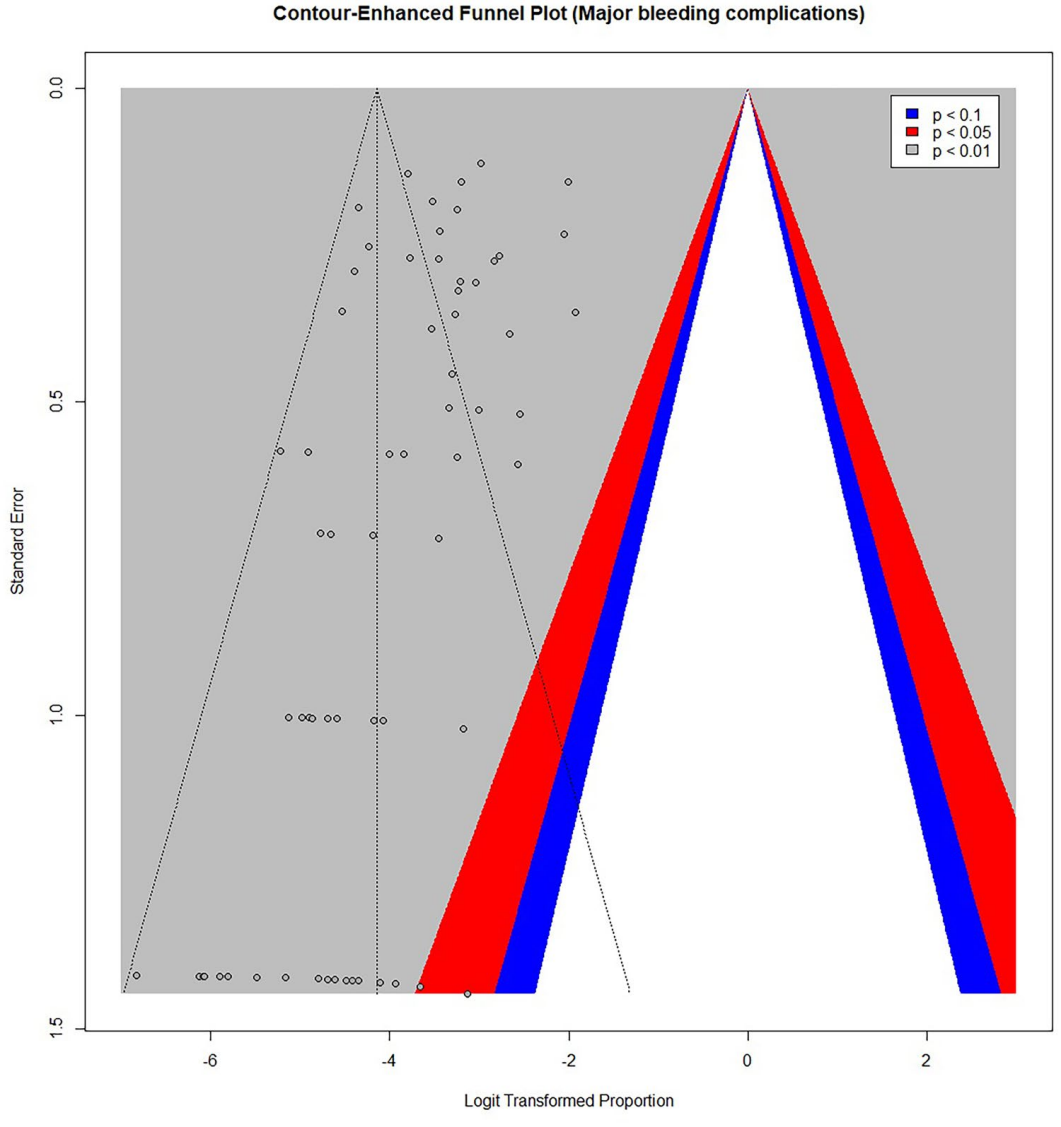
Funnel plot assessing potential publication bias in reported major bleeding complication rates. Each point represents an individual study. The x-axis shows the logit-transformed proportion of major complications and the y-axis the standard error. The dashed vertical line represents the pooled estimate. The shaded regions indicate significance contours (*p* < 0.10, *p* < 0.05, *p* < 0.01). Asymmetry in the distribution of studies may suggest potential publication bias.

Between-study heterogeneity was substantial (I^2^ = 75.8%, 95% CI: 69.5 to 80.8), indicating considerable variability beyond sampling error. Funnel plot inspection (Figure 3) showed asymmetry, and Egger’s test suggested potential publication bias with a significant intercept of −1.36 (SE 0.37, t = −3.64, df = 66, *p* = 0.0005).

### Minor complications and total complications

The pooled estimate of macroscopic hematuria was 2.66% (95% CI: 1.64% to 4.28%) for 16 G and 2.54% (95% CI: 1.06% to 5.98%) for 18 G needles. Needle size did not significantly affect hematuria risk (estimate difference 0.12; 95% CI: −3.68% to 3.92%) (Supplementary; Figure S8). Univariable meta-regression showed a significant correlation between study biopsy number and hematuria risk, indicating that smaller studies predicted higher hematuria rates (Supplementary; Table S9). No other variables were significantly associated.

Detection of hematomas varied between studies. Some reported routine ultrasounds immediately, 6–8 h, or the next morning after biopsy, while others only scanned if clinically indicated. This led to a wide range of hematoma rates from 0% to 57.8%, complicating comparisons. The pooled hematoma estimate was 5.06% (95% CI: 2.70% to 9.30%) for 16 G and 8.28% (95% CI: 5.08% to 13.21%) for 18 G needles. Needle size did not significantly affect hematoma rates (estimate difference −3.22; 95% CI: −9.73% to 3.28%) (Supplementary; Figure S9).

The pooled estimate of all complications (hematomas, macroscopic hematuria, transfusion, embolization, nephrectomy, surgery, and death) was 9.15% (95% CI: 6.23% to 13.26%) for 16 G and 7.62% (95% CI: 4.80% to 11.91%) for 18 G needles. Needle size did not significantly affect total complication rates (estimate difference 1.53%; 95% CI: −4.41% to 7.47%) (Supplementary; Figure S10).

### Effectiveness of the two needle sizes

**Figure 4.**
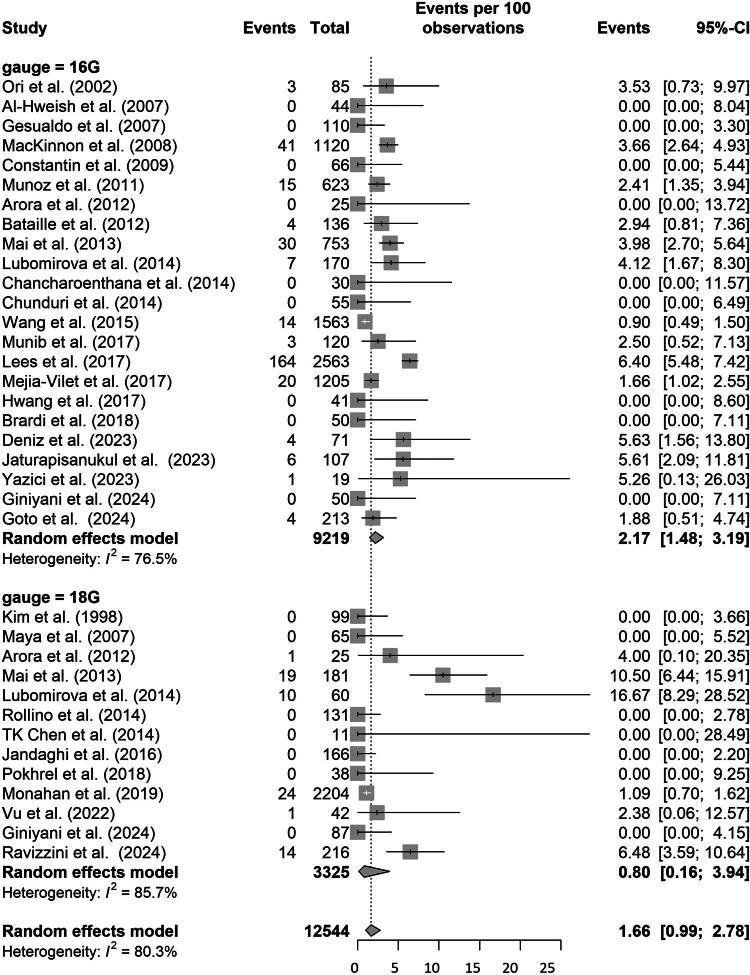
Proportion of inconclusive biopsies stratified by needle size. Random effects model testing difference between the two needle sizes and the estimate of inconclusive biopsies. CI, Confidence Interval. I^2^, Heterogeneity.

Among the reviewed studies, 32 reported data on inconclusive biopsies, with varying definitions. Some defined inconclusiveness by a glomeruli-count below a threshold, while others lacked clear criteria. Numerous studies reflected on the diagnostic implications, noting that certain kidney diseases could be diagnosed with a single glomerulus, while others required examination of a minimum of 10 glomeruli. Given this heterogeneity in outcome definition, pooled estimates should be interpreted cautiously. The estimated inconclusive biopsy rate was 2.17% (95% CI: 1.48% to 3.19%) for 16 G and 0.80% (95% CI: 0.16% to 3.94%) for 18 G needles. There was no significant difference between needle sizes (estimate difference 1.37%; 95% CI: −1.93% to 4.67%) (Figure 4).

The number of glomeruli was reported in 29 studies, but reporting methods varied considerably. Some studies provided mean counts specific to biopsy cores examined by light microscopy, others included both light microscopy and immunofluorescence, and some reported counts across all processed tissue. Several studies did not specify the methodological basis of the reported counts.

Because of these inconsistencies in glomerular count definitions and reporting practices, direct comparison across studies is limited. Using Welch’s t-test across the 29 studies showed no statistically significant difference between needle sizes (Table 3).

**Table 3.** Number of glomeruli per biopsy according to reporting method in the included studies.

	18G		16G		Difference (95% CI)
	Study arms (n)	Mean number of glomeruli (SD)	Study arms (n)	Mean number of glomeruli (SD)	
LM core	2	9.7 (2.9)	7	13.6 (3.7)	3.9 (−6.3; 14.0)
LM + IF cores	2	19.4 (1.9)	2	18.4 (1.0)	−1.0 (−10.2; 8.2)
All tissue	8	17.5 (6.5)	11	18.8 (5.2)	1.3 (−4.7; 7.3)
Not reported	2	12.7 (1.0)	0	NA	NA

Reported mean glomerular counts varied depending on whether studies reported counts from light microscopy cores only (LM), combined light microscopy and immunofluorescence cores (LM + IF), or all processed tissue. Because of these differences in reporting methodology, direct comparison across studies should be interpreted cautiously.

Differences were estimated using Welch’s t-test comparing study-level mean glomerular counts.

CI, confidence interval. EM, electron microscopy. G, gauge. IF, immunofluorescence. LM, light microscopy. SD, standard deviation.

Among the six studies directly comparing both needle sizes, five reported mean glomerular counts. The mean was 14.4 (SD 8.5) for 18 G and 16.4 (SD 5.9) for 16 G biopsies, with no statistically significant difference (1.99 [95% CI: −12.94 to 8.96]; *p* = 0.68).

## Discussion

In this systematic review and meta-analysis of 62 studies comprising 22 208 adult native kidney biopsies performed under real-time ultrasound guidance, we evaluated complication rates and diagnostic outcomes reported in studies using either 16 G or 18 G needles. The pooled major complication rate was numerically higher in studies using 16 G needles (1.83%) compared with 18 G needles (1.29%). Statistical analyses did not reveal a significant difference between needle sizes. However, because most included studies were single-arm cohorts, comparisons between needle sizes rely on indirect between-study contrasts and should therefore be interpreted with caution.

No significant differences were observed in minor complications or inconclusive biopsy rates. However, interpretation of inconclusive biopsy rates and glomerular yield is limited by substantial variation in definitions and reporting practices across studies, which limits direct comparability.

In study-level meta-regression analyses, studies with a higher prevalence of AKI, lower mean eGFR, and lower mean hemoglobin reported higher pooled major complication rates. Funnel plot asymmetry and Egger’s test suggested potential publication bias.

To our knowledge, this is the first systematic review and meta-analysis focusing exclusively on real-time ultrasound-guided native kidney biopsies in adults and reporting complication rates according to needle gauge.

While previous reviews primarily examined overall bleeding complications and multiple contributing factors [9,89], our study focused specifically on needle size and major complications, which guided our inclusion criteria. In line with Poggio et al. [89], Corapi et al. [9], and Kajawo et al. [90], we found no significant difference in complication rates between 16 G and 18 G needles. This aligns with other large cohort studies, which—although not eligible for inclusion due to mixed populations or inseparable data—also reported no substantial impact of needle size on complication rates [3,91,92].

A recent review by Zhan and Lou [93] primarily examined glomerular yield between needle sizes but also reported major complication rates. A key strength was the inclusion of studies only using both 16 G and 18 G needles, enabling direct comparison. However, the inclusion of mixed cohorts (e.g. pediatric and adult populations, graft and native biopsies) likely increased heterogeneity and limited generalizability [3,94]. Their analysis was also based on ∼3 000 biopsies, compared to over 22 000 in our study. They found that 16 G needles yielded significantly more glomeruli, with no significant difference in major complications.

Major post-biopsy complications—defined as transfusion, embolization, surgery, nephrectomy, and/or death—are typically evident due to their symptomatic presentation. If not explicitly reported in the reviewed articles, they were omitted from our analysis. We are therefore confident that our primary outcome was consistently assessed. However, thresholds for interventions such as transfusion, as well as imaging practices and post-biopsy observation protocols, vary across centers. In retrospective cohorts, detection and reporting of complications may therefore depend partly on local clinical practice and documentation, which may contribute to variability in reported complication rates across studies. Minor complications may be underreported in retrospective studies, especially in clinically stable patients who may not undergo routine imaging or prolonged observation. The relevance of asymptomatic hematomas and microscopic hematuria remains debated, though current evidence suggests limited immediate clinical impact [2,95].

Studies including a higher prevalence of AKI, lower mean eGFR, and lower mean hemoglobin reported higher pooled complication rates, consistent with known clinical risk factors [9]. We used a Welch t-test to compare reported mean glomerular counts between needle sizes. This comparison is limited by differences in how glomerular counts were reported across studies and should therefore be interpreted cautiously. At the study level, smaller studies were associated with higher reported rates of macroscopic hematuria, suggesting that studies with fewer biopsies may report more complications—possibly due to publication bias.

Operator specialty (nephrologist vs radiologist) may influence biopsy practice patterns and complication rates. In our study-level meta-regression, operator specialty was not associated with major complication rates. However, information on operator specialty was not consistently reported across studies, and confounding cannot be excluded.

### Limitations

This systematic review has several important limitations. First, most included studies were retrospective single-arm cohorts, limiting data quality and making direct head-to-head comparisons difficult. Consequently, any observed differences between 16 G and 18 G needles reflect indirect between-study contrasts and cannot be interpreted as causal.

Second, between-study heterogeneity was moderate to substantial (I^2^ 73–80%), indicating considerable variability beyond sampling error. This substantial heterogeneity suggests that a single pooled estimate may oversimplify the true risk and may not reflect complication rates in any “typical” clinical setting. It likely reflects differences in patient characteristics, procedural factors, center-level practices, and study inclusion criteria. For example, some studies excluded patients with conditions such as uncontrolled hypertension [31,39,78], while others focused on specific subgroups, including outpatients [74,75], elderly patients [42,81], or individuals with certain diseases [68,79,80].

Third, 12 studies were excluded due to language restrictions. While this was necessary for feasibility, it may have introduced selection bias if these studies differed systematically from included studies, potentially affecting the generalizability of our findings.

Fourth, publication bias may have affected the findings, as suggested by funnel plot asymmetry and a significant Egger’s test. However, these tools were developed for comparative meta-analyses and may not be fully valid for proportional data, where “positive” results are less clearly defined. Therefore, evidence of publication bias should be interpreted with caution and the true extent of reporting bias remains uncertain. Furthermore, centers with higher complication rates may be less likely to publish, potentially underestimating true risk.

Fifth, selection bias may have influenced reported complication rates. High-risk biopsies could have been performed using different needle sizes, fewer passes, or altered protocols, which may confound indirect comparisons.

Sixth, meta-regression analyses were performed at the study level: Studies with a higher prevalence of AKI, lower mean eGFR, or lower mean hemoglobin tended to report higher pooled major complication rates. However, because these analyses are based on study-level data, they cannot determine whether these factors represent individual patient risk. The analyses were also univariable and may therefore be influenced by confounding. These findings should be considered hypothesis-generating.

Finally, the retrospective design of most studies, substantial heterogeneity, indirect comparisons, and language restrictions limit the strength and generalizability of our findings.

### Perspectives

Future research should address the remaining uncertainties identified in our study. Large, prospective studies with standardized protocols are needed to strengthen the evidence on optimal needle size. Ideally, a randomized controlled trial assigning patients to biopsy with either a 16 G or 18 G needle should be conducted. Based on current findings, such a trial would be ethically feasible and could offer conclusive guidance on needle gauge selection for native kidney biopsies.

## Conclusion

In conclusion, this systematic review and meta-analysis found low reported major complication rates for both 16 G and 18 G needles in adult native kidney biopsies. Pooled complication rates were numerically higher in studies using 16 G needles, but statistical analyses did not demonstrate a significant difference. As most included studies were single-arm cohorts, comparisons between needle sizes rely on indirect between-study comparisons and should be interpreted with caution. Studies including a higher proportion of patients with low eGFR, AKI, or low hemoglobin reported higher pooled complication rates. Well-designed prospective comparative studies (ideally randomized controlled trials) are needed to determine the optimal needle size and enhance biopsy safety and effectiveness.

## Supplementary Material

Supplementary tables and figures.docx

## Data Availability

The dataset and analysis codes will be available from the corresponding author upon reasonable request.
