# Supplementary material for: Complication rates of 16- and 18-gauge needles for native kidney biopsies: a systematic review and proportional meta-analysis
Source: Ren Fail. 2026 May 25;48(1):2665032. doi: 10.1080/0886022X.2026.2665032 (PMC13202671; doi:10.1080/0886022X.2026.2665032)
Supplement: Supplementary tables and figures.docx [file IRNF_A_2665032_SM0839.docx]

**Supplementary**

[Table S1 PRISMA 2020 Checklist 2](#_Toc224338595)

[Table S2 Search String conducted in the Database Ovid MEDLINE 5](#_Toc224338596)

[Table S3 Search String conducted in the Database CENTRAL 6](#_Toc224338597)

[Table S4 Search String conducted in the Database EMBASE 7](#_Toc224338598)

[Table S5 Articles excluded due to language barrier 9](#_Toc224338599)

[RoB for cohort studies. 10](#_Toc224338600)

[Figure S1 The risk of bias assessment of cohort studies in accordance with the Joanna Briggs Institute critical appraisal tool for cohort studies 11](#_Toc224338601)

[RoB for RCT. 13](#_Toc224338602)

[Figure S2 The risk of bias assessment of randomized controlled trials in accordance with the Joanna Briggs Institute critical appraisal tool for cohort studies 14](#_Toc224338603)

[Table S6 Characteristics of included studies 15](#_Toc224338604)

[Figure S3 Proportion of major complications stratified by needle size for studies with high quality 19](#_Toc224338605)

[Figure S4 Risk ratio of major complications stratified by needle size 19](#_Toc224338606)

[Figure S5 Proportion of transfusion by needle size 20](#_Toc224338607)

[Figure S6 Proportion of embolization by needle size 21](#_Toc224338608)

[Figure S7 Proportion of death stratified by needle size 22](#_Toc224338609)

[Figure S8 Proportion of macroscopic haematuria stratified by needle size 23](#_Toc224338610)

[Figure S9 Proportion of hematomas stratified by needle size 24](#_Toc224338611)

[Figure S10 Proportion of total complications (haematomas, macroscopic haematuria, transfusion, embolization, nephrectomy, other surgery, and/or death) stratified by gauge size 25](#_Toc224338612)

[Table S7 Univariable meta-regression analysis for major complications 26](#_Toc224338613)

[Table S8 Univariable meta-regression analysis for transfusion 26](#_Toc224338614)

[Table S9 Univariable meta-regression analysis for macroscopic haematuria 27](#_Toc224338615)

| Table S1 PRISMA 2020 Checklist | | | |
| --- | --- | --- | --- |
| **Section and Topic** | **Item #** | **Checklist item** | **Location where item is reported** |
| **TITLE** | | |  |
| Title | 1 | Identify the report as a systematic review. | Page 1 |
| **ABSTRACT** | | |  |
| Abstract | 2 | See the PRISMA 2020 for Abstracts checklist. | Page 4 |
| **INTRODUCTION** | | |  |
| Rationale | 3 | Describe the rationale for the review in the context of existing knowledge. | Page 5 |
| Objectives | 4 | Provide an explicit statement of the objective(s) or question(s) the review addresses. | Page 5/6 |
| **METHODS** | | |  |
| Eligibility criteria | 5 | Specify the inclusion and exclusion criteria for the review and how studies were grouped for the syntheses. | Page 6 |
| Information sources | 6 | Specify all databases, registers, websites, organisations, reference lists and other sources searched or consulted to identify studies. Specify the date when each source was last searched or consulted. | Page 6 |
| Search strategy | 7 | Present the full search strategies for all databases, registers and websites, including any filters and limits used. | Supplementary p 2-4 |
| Selection process | 8 | Specify the methods used to decide whether a study met the inclusion criteria of the review, including how many reviewers screened each record and each report retrieved, whether they worked independently, and if applicable, details of automation tools used in the process. | Page 7 |
| Data collection process | 9 | Specify the methods used to collect data from reports, including how many reviewers collected data from each report, whether they worked independently, any processes for obtaining or confirming data from study investigators, and if applicable, details of automation tools used in the process. | Page 7 |
| Data items | 10a | List and define all outcomes for which data were sought. Specify whether all results that were compatible with each outcome domain in each study were sought (e.g. for all measures, time points, analyses), and if not, the methods used to decide which results to collect. | Page 7 |
|  | 10b | List and define all other variables for which data were sought (e.g. participant and intervention characteristics, funding sources). Describe any assumptions made about any missing or unclear information. | Page 7 |
| Study risk of bias assessment | 11 | Specify the methods used to assess risk of bias in the included studies, including details of the tool(s) used, how many reviewers assessed each study and whether they worked independently, and if applicable, details of automation tools used in the process. | Page 8 and supplementary page 7+10 |
| Effect measures | 12 | Specify for each outcome the effect measure(s) (e.g. risk ratio, mean difference) used in the synthesis or presentation of results. | Page 8 |
| Synthesis methods | 13a | Describe the processes used to decide which studies were eligible for each synthesis (e.g. tabulating the study intervention characteristics and comparing against the planned groups for each synthesis (item #5)). | Page 8 |
|  | 13b | Describe any methods required to prepare the data for presentation or synthesis, such as handling of missing summary statistics, or data conversions. | Page 8 |
|  | 13c | Describe any methods used to tabulate or visually display results of individual studies and syntheses. | Page 8 |
|  | 13d | Describe any methods used to synthesize results and provide a rationale for the choice(s). If meta-analysis was performed, describe the model(s), method(s) to identify the presence and extent of statistical heterogeneity, and software package(s) used. | Page 8 + 9 |
|  | 13e | Describe any methods used to explore possible causes of heterogeneity among study results (e.g. subgroup analysis, meta-regression). | Page 9 |
|  | 13f | Describe any sensitivity analyses conducted to assess robustness of the synthesized results. | Page 9 |
| Reporting bias assessment | 14 | Describe any methods used to assess risk of bias due to missing results in a synthesis (arising from reporting biases). | Page 9 |
| Certainty assessment | 15 | Describe any methods used to assess certainty (or confidence) in the body of evidence for an outcome. | Page 8/9 |
| **RESULTS** | | |  |
| Study selection | 16a | Describe the results of the search and selection process, from the number of records identified in the search to the number of studies included in the review, ideally using a flow diagram. | Page 10 |
|  | 16b | Cite studies that might appear to meet the inclusion criteria, but which were excluded, and explain why they were excluded. | Page 19 and supplementary page 6 |
| Study characteristics | 17 | Cite each included study and present its characteristics. | Page 11+12 |
| Risk of bias in studies | 18 | Present assessments of risk of bias for each included study. | Page 10 and supplementary page 7-11 |
| Results of individual studies | 19 | For all outcomes, present, for each study: (a) summary statistics for each group (where appropriate) and (b) an effect estimate and its precision (e.g. confidence/credible interval), ideally using structured tables or plots. | Page 13 and Figure 2 |
| Results of syntheses | 20a | For each synthesis, briefly summarise the characteristics and risk of bias among contributing studies. | Page 13+14+15+16 |
|  | 20b | Present results of all statistical syntheses conducted. If meta-analysis was done, present for each the summary estimate and its precision (e.g. confidence/credible interval) and measures of statistical heterogeneity. If comparing groups, describe the direction of the effect. | Page 14+15+16 Figure 4 and figure S3 S4 S5 S6 S7 S8 S9 and S10 |
|  | 20c | Present results of all investigations of possible causes of heterogeneity among study results. | Page 13+14+15 and Table 5s 6s and 7s |
|  | 20d | Present results of all sensitivity analyses conducted to assess the robustness of the synthesized results. | Page 13 + 16 and figure S3 and S4 |
| Reporting biases | 21 | Present assessments of risk of bias due to missing results (arising from reporting biases) for each synthesis assessed. | Page 14 and figure 3 |
| Certainty of evidence | 22 | Present assessments of certainty (or confidence) in the body of evidence for each outcome assessed. | Page 10 |
| **DISCUSSION** | | |  |
| Discussion | 23a | Provide a general interpretation of the results in the context of other evidence. | Page 17+18 |
|  | 23b | Discuss any limitations of the evidence included in the review. | Page 19 |
|  | 23c | Discuss any limitations of the review processes used. | Page 19 |
|  | 23d | Discuss implications of the results for practice, policy, and future research. | Page 19 +20 |
| **OTHER INFORMATION** | | |  |
| Registration and protocol | 24a | Provide registration information for the review, including register name and registration number, or state that the review was not registered. | Page 6 |
|  | 24b | Indicate where the review protocol can be accessed, or state that a protocol was not prepared. | Page 6 |
|  | 24c | Describe and explain any amendments to information provided at registration or in the protocol. | N/A |
| Support | 25 | Describe sources of financial or non-financial support for the review, and the role of the funders or sponsors in the review. | Page 21 |
| Competing interests | 26 | Declare any competing interests of review authors. | Page 20+21 |
| Availability of data, code and other materials | 27 | Report which of the following are publicly available and where they can be found: template data collection forms; data extracted from included studies; data used for all analyses; analytic code; any other materials used in the review. | Page 20 |

*From:*  Page MJ, McKenzie JE, Bossuyt PM, Boutron I, Hoffmann TC, Mulrow CD, et al. The PRISMA 2020 statement: an updated guideline for reporting systematic reviews. BMJ 2021;372:n71. doi: 10.1136/bmj.n71

For more information, visit: <http://www.prisma-statement.org/>

| ****Table S2 Search String conducted in the Database Ovid MEDLINE**** | | |
| --- | --- | --- |
| # | Searches | Results |
| 1 | exp Kidney/ | 372818 |
| 2 | exp Kidney Diseases/ | 567624 |
| 3 | 1 or 2 | 813164 |
| 4 | Biopsy/ | 187040 |
| 5 | Biopsy, Needle/ | 49731 |
| 6 | Needles/ | 17204 |
| 7 | or/4-6 | 251177 |
| 8 | 3 and 7 | 21066 |
| 9 | ((kidney$ or nephro$ or renal) adj4 (biops$ or needle$)).ti,ab,kf,kw. | 29103 |
| 10 | 8 or 9 | 40527 |
| 11 | Hemorrhage/ | 81009 |
| 12 | Blood Loss, Surgical/ | 20109 |
| 13 | Hematuria/ | 12634 |
| 14 | Biopsy/ae, co | 3749 |
| 15 | Biopsy, Needle/ae | 3303 |
| 16 | Needles/ae | 1144 |
| 17 | (((side or adverse) adj effect$) or adverse event$ or bleeding$ or complication$).ti,ab,kf,kw. | 1935820 |
| 18 | or/11-17 | 2001993 |
| 19 | 10 and 18 | 6850 |
| 20 | randomi#ed controlled trial.pt. | 588000 |
| 21 | controlled clinical trial.pt. | 95208 |
| 22 | randomi#ed.ab. | 710333 |
| 23 | placebo.ab. | 236252 |
| 24 | drug therapy.fs. | 2569922 |
| 25 | randomly.ab. | 403195 |
| 26 | trial.ab. | 638152 |
| 27 | groups.ab. | 2484285 |
| 28 | or/20-27 | 5615392 |
| 29 | exp Cohort Studies/ | 2453908 |
| 30 | Risk Factors/ | 946328 |
| 31 | "Surveys and Questionnaires"/ | 555131 |
| 32 | (cohort$ or longitudinal$ or observation$ or population-based or prospective$ or retrospective$).ti,ab,kf,kw. | 3360698 |
| 33 | or/29-32 | 5193780 |
| 34 | 28 or 33 | 9292815 |
| 35 | 19 and 34 | 3397 |
| 36 | (exp Infant/ or exp Child/ or Adolescent/) not exp Adult/ | 2113553 |
| 37 | 35 not 36 | 2900 |
| 38 | exp Animals/ not humans.sh. | 5098507 |
| 39 | 37 not 38 | 2838 |

| ****Table S3 Search String conducted in the Database CENTRAL**** | |
| --- | --- |
| ID Search  #1 [mh Kidney]  #2 [mh "Kidney Diseases"]  #3 #1 OR #2  #4 [mh ^Biopsy]  #5 [mh "Biopsy, Needle"]  #6 [mh ^Needles]  #7 #4 OR #5 OR #6  #8 #3 AND #7  #9 ((kidney* OR nephro* OR renal) NEAR/4 (biops* OR needle?)):ti,ab,kw  #10 #8 OR #9  #11 [mh ^Hemorrhage]  #12 [mh ^"Blood Loss, Surgical"]  #13 [mh ^Hematuria]  #14 (adverse NEXT effect* OR side NEXT effect* OR adverse NEXT event* OR bleeding* OR complication*):ti,ab,kw  #15 #11 OR #12 OR #13 OR #14  #16 #10 AND #15  #17 (([mh Infant] or [mh Child] or [mh Adolescent]) not [mh Adult])  #18 #16 not #17  #19 ([mh Animals] not [mh humans])  #20 #18 not #19  #21 #20 in Trials | Hits  4723  20332  23304  3754  1413  1398  6415  304  1558  1729  5116  3073  207  554663  555964  866  74966  835  2686  833  830 |

| Table S4 Search String conducted in the Database EMBASE | | |
| --- | --- | --- |
| **#** | **Searches** | **Results** |
| 1 | exp kidney/ | 434964 |
| 2 | exp kidney diseases/ | 1127880 |
| 3 | 1 or 2 | 1389035 |
| 4 | biopsy/ | 177464 |
| 5 | biopsy needle/ | 2837 |
| 6 | needle/ | 49644 |
| 7 | or/4-6 | 227198 |
| 8 | 3 and 7 | 18134 |
| 9 | kidney biopsy/ | 55898 |
| 10 | ((kidney$ or nephro$ or renal) adj4 (biops$ or needle$)).ti,ab,kf,kw. | 48735 |
| 11 | or/8-10 | 83304 |
| 12 | bleeding/ | 338478 |
| 13 | exsanguination/ | 1351 |
| 14 | exp hematoma/ | 121963 |
| 15 | operative blood loss/ | 35919 |
| 16 | postoperative hemorrhage/ | 45467 |
| 17 | biopsy/ae [Adverse Drug Reaction] | 546 |
| 18 | biopsy needle/am, dc [Adverse Device Effect, Device Comparison] | 86 |
| 19 | needle/am, ae, dc [Adverse Device Effect, Adverse Drug Reaction, Device Comparison] | 461 |
| 20 | (((side or adverse) adj effect$) or adverse event$ or bleeding$ or complication$).ti,ab,kf,kw. | 2847257 |
| 21 | or/12-20 | 3053502 |
| 22 | 11 and 21 | 14865 |
| 23 | randomized controlled trial/ | 773510 |
| 24 | controlled clinical study/ | 468720 |
| 25 | random$.ti,ab. | 1936999 |
| 26 | randomization/ | 98364 |
| 27 | intermethod comparison/ | 294720 |
| 28 | placebo.ti,ab. | 361852 |
| 29 | (compare or compared or comparison).ti. | 597314 |
| 30 | ((evaluated or evaluate or evaluating or assessed or assess) and (compare or compared or comparing or comparison)).ab. | 2719515 |
| 31 | (open adj label).ti,ab. | 107091 |
| 32 | ((double or single or doubly or singly) adj (blind or blinded or blindly)).ti,ab. | 271513 |
| 33 | double blind procedure/ | 207957 |
| 34 | parallel group$1.ti,ab. | 31734 |
| 35 | (crossover or cross over).ti,ab. | 123036 |
| 36 | ((assign$ or match or matched or allocation) adj5 (alternate or group$1 or intervention$1 or patient$1 or subject$1 or participant$1)).ti,ab. | 409002 |
| 37 | (assigned or allocated).ti,ab. | 481769 |
| 38 | (controlled adj7 (study or design or trial)).ti,ab. | 443808 |
| 39 | (volunteer or volunteers).ti,ab. | 279771 |
| 40 | human experiment/ | 640145 |
| 41 | trial.ti. | 395774 |
| 42 | or/23-41 | 6218282 |
| 43 | cohort analysis/ | 1003115 |
| 44 | risk factor/ | 1305411 |
| 45 | questionnaire/ | 875352 |
| 46 | (cohort$ or longitudinal$ or observation$ or population-based or prospective$ or retrospective$).ti,ab,kf,kw. | 5056200 |
| 47 | or/43-46 | 6642992 |
| 48 | 42 or 47 | 10912681 |
| 49 | 22 and 48 | 6008 |
| 50 | (rat or rats or mouse or mice or swine or porcine or murine or sheep or lambs or pigs or piglets or rabbit or rabbits or cat or cats or dog or dogs or cattle or bovine or monkey or monkeys or trout or marmoset$1).ti. and animal experiment/ | 1215563 |
| 51 | animal experiment/ not (human experiment/ or human/) | 2552479 |
| 52 | or/50-51 | 2619578 |
| 53 | 49 not 52 | 5943 |
| 54 | exp juvenile/ not exp adult/ | 2544455 |
| 55 | 53 not 54 | 5410 |
| 56 | limit 55 to exclude medline journals | 645 |

| ****Table S5 Articles excluded due to language barrier**** |
| --- |
| 1. Aimino M et al. [Percutaneous renal biopsy with a semi-automate device]. Minerva Urol Nefrol. 2001 Jun;53(2):65-7. 2. Campobasso N et al. Echography in the diagnosis and follow-up of renal biopsy complications]. Arch Ital Urol Androl. 1997 Jun;69(3):193-9. 3. Fortuño Andrés JR et al. [Safety and yield of percutaneous renal biopsy using an automatic 16G needle in native kidneys]. Radiologia. 2010 Mar-Apr;52(2):153-6. 4. Fukunaga S et al. [Investigation of the safety and usefulness of a renal biopsy for older elderly patients ≥75 years old]. Nihon Ronen Igakkai Zasshi. 2021;58(3):453-458. 5. González-Michaca L et al. [Percutaneous kidney biopsy, analysis of 26 years: complication rate and risk factors; comment]. Rev Invest Clin. 2000 Mar-Apr;52(2):125-31. 6. Benito MH et al. Indications, histopathological results and safety of percutaneous renal biopsy: A comparative study between elderly (65-79 years) and very elderly patients (80 years or more). Diálisis y trasplante. Vol. 34, Nº. 4, 2013, pages 166-170 7. Iannaccone S et al. [Renal biopsy: outpatient procedure?]. G Ital Nefrol. 2003 May-Jun;20(3):253-7. 8. Koumoto J. [Complications of percutaneous renal biopsy]. Nihon Jinzo Gakkai Shi. 1992 Jul;34(7):789-99. 9. Mhamedi SA et al. La ponction biopsie rénale: indications, complications et résultats. Pan Afr Med J. 2018 Sep 20;31:44. 10. Quattrocchio G et al. [Biopsy experience at the G. Bosco Hospital from 1996 to 1999]. Minerva Urol Nefrol. 2001 Jun;53(2):81-6. 11. Tao JL et al. [Risk factors of post-renal biopsy bleeding]. Zhongguo Yi Xue Ke Xue Yuan Xue Bao. 2008 Jun;30(3):313-7. 12. Zhang L et al. [Analysis of bleeding risk in percutaneous renal biopsy in Tibet]. Beijing Da Xue Xue Bao Yi Xue Ban. 2021 Mar 11;53(2):298-301. |
| Articles in languages other than English, German, Danish, Swedish, or Norwegian were excluded from the synthesis due to language barriers, despite being eligible based on title and abstract. Based on this criterion we excluded 12 articles, and they are listed here for readers who wish to read and analyse. |

## RoB for cohort studies.

For the risk of bias (RoB) assessment of included cohort studies we used the Joanna Briggs Institute critical appraisal tool for cohort studies.

The primary outcome is major complications defined as blood transfusion, surgical intervention, or death. All answers in the tool regarding outcome was assessed based on these parameters.

The outcome was considered reported in a valid and reliable way, in all cases, where missing data wasn’t reported. A major bleeding event will in all cases be detected because of the clinical severe symptoms.

Follow-up-time was considered sufficient, if the reported follow-up time was stated to be = or > six hours. Most major complications occur within this window. If not, the patient will be readmitted, and the event will be recorded.

Major complications after a kidney biopsy require hospital care and intervention, and therefore it will be registered in the medical records. Therefore, follow up was considered complete unless the paper stated otherwise.

If major outcomes couldn’t be assessed the article was excluded.

In the JBI RoB tool for cohort studies there are 11 domains. To each domain the reviewer can score Yes, No, Unclear and Not Applicable. The answer Not Applicable and No was scored 0. The answer Unclear scored 1, and the answer yes scored 2. With the 11 domains this gave a possible maximum score of 22

Based on the scoring system the articles were categorized as low (score ≤ 11), moderate (score 12-16), and high (≥ 17).

| Figure S1 The risk of bias assessment of cohort studies in accordance with the Joanna Briggs Institute critical appraisal tool for cohort studies |
| --- |
| 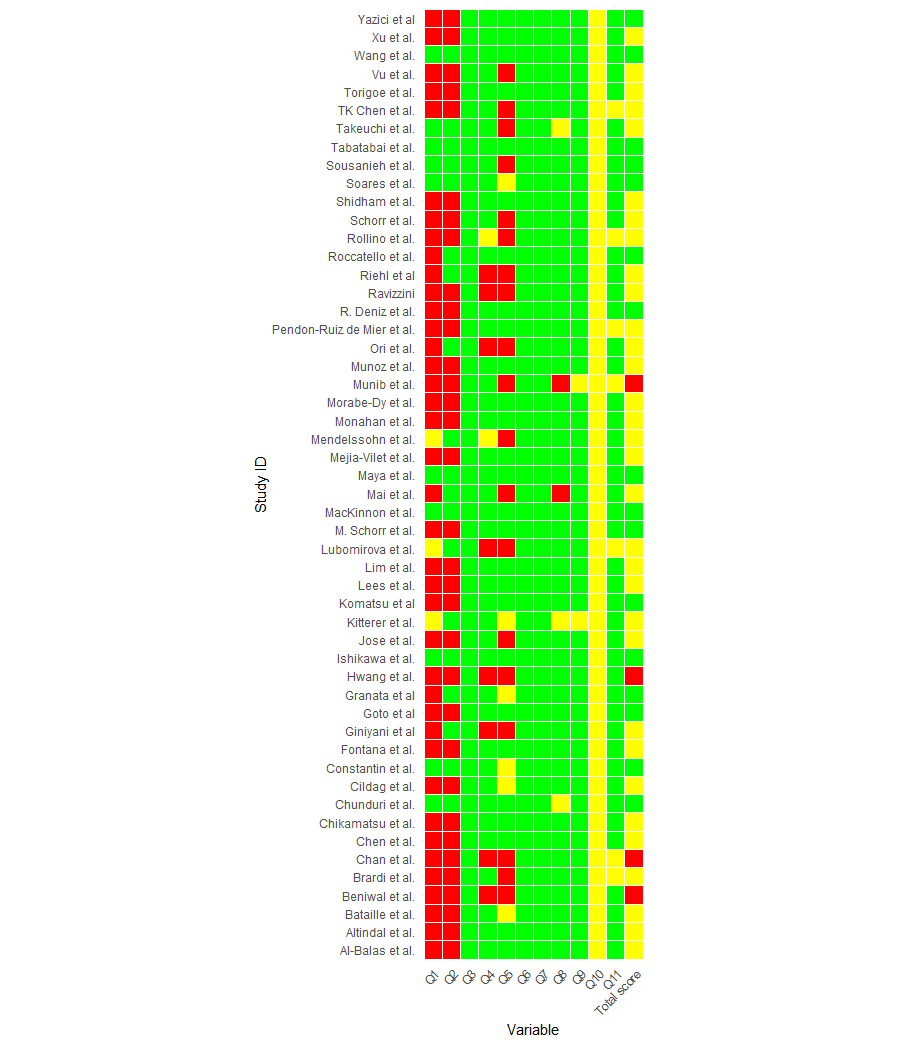 |
| Risk of bias assessment of included cohort studies using the Joanna Briggs Institute (JBI) critical appraisal tool. The tool contains 11 domains (Q1–Q11) assessing study design, exposure measurement, confounding, outcome assessment, follow-up, and statistical analysis.  Each domain was scored as follows: Yes = 2, Unclear = 1, No or Not applicable = 0, resulting in a maximum possible score of 22. Based on the total score, studies were categorized as low quality (≤11 points), moderate quality (12–16 points), or high quality (≥17 points).  Color coding in the figure represents the overall quality category: red = low, yellow = moderate, green = high.  Q1: Were the two groups similar and recruited from the same population?  Q2: Were the exposures measured similarly to assign people to both exposed and unexposed groups?  Q3: Was the exposure measured in a valid and reliable way?  Q4: Were confounding factors identified?  Q5: Were strategies to deal with confounding factors stated?  Q6: Were the groups/participants free of the outcome at the start of the study (or at the moment of exposure)?  Q7: Were the outcomes measured in a valid and reliable way?  Q8: Was the follow up time reported and sufficient to be long enough for outcomes to occur?  Q9: Was follow up complete, and if not, were the reasons to loss to follow up described and explored?  Q10: Were strategies to address incomplete follow up utilized?  Q11: Was appropriate statistical analysis used? |

## RoB for RCT.

For the RoB assessment of included RCT’s we used the Joanna Briggs Institute critical appraisal tool for RCT studies 12,13.

The primary outcome is major complications defined as blood transfusion, surgical intervention, or death. All answers in the tool regarding outcome was assessed based on these parameters.

The outcome was considered reported in a valid and reliable way, in all cases, where missing data wasn’t reported. A major bleeding event will in all cases be detected because of the clinical severe symptoms.

Follow-up-time was considered sufficient if the reported follow-up time was stated to be = or > six hours. Most major complications occur within this window. If not, the patient will be readmitted, and the event will be recorded.

Major complications after a kidney biopsy require hospital care and intervention, and therefore it will be registered in the medical records. Therefore, follow up was considered complete unless the paper stated otherwise.

If major outcomes couldn’t be assessed the article was excluded.

In the JBI RoB tool for RCT’s there are 13 questions. To each question the reviewer can score “Yes”, “No”, “Unclear” and “Not Applicable”. The answer “Not Applicable” and “No” was scored 0. The answer “Unclear” scored 1, and the answer “Yes” scored 2. With 13 questions this gave a possible maximum score of 26. Based on the scoring system the articles quality were categorized as low (score ≤ 15), moderate (score 16-21), and high (≥ 22).

| Figure S2 The risk of bias assessment of randomized controlled trials in accordance with the Joanna Briggs Institute critical appraisal tool for cohort studies |
| --- |
| 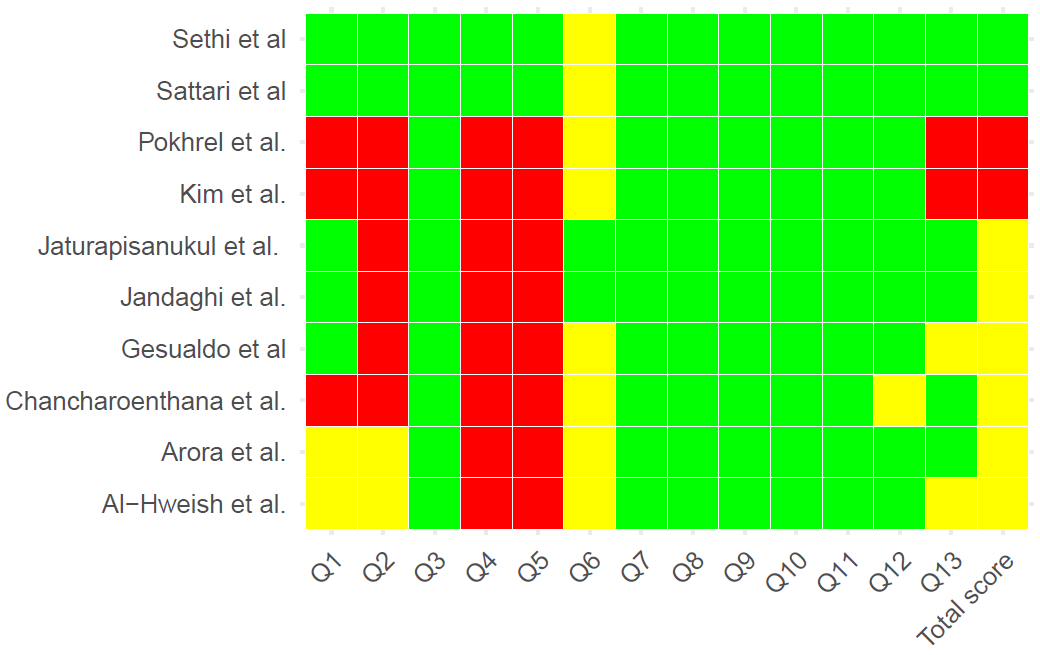 |
| Risk of bias assessment of included cohort studies using the Joanna Briggs Institute (JBI) critical appraisal tool. The tool contains 11 domains (Q1–Q11) assessing study design, exposure measurement, confounding, outcome assessment, follow-up, and statistical analysis.  Each domain was scored as follows: Yes = 2, Unclear = 1, No or Not applicable = 0, resulting in a maximum possible score of 22. Based on the total score, studies were categorized as low quality (≤11 points), moderate quality (12–16 points), or high quality (≥17 points).  Color coding in the figure represents the overall quality category: red = low, yellow = moderate, green = high.  Q1: Was true randomization used for assignment of participants to treatment groups?  Q2: Was allocation to treatment groups concealed?  Q3: Were treatment groups similar at the baseline?  Q4: Were participants blind to treatment assignment?  Q5: Were those delivering treatment blind to treatment assignment?  Q6: Were outcomes assessors blind to treatment assignment?  Q7: Were treatment groups treated identically other than the intervention of interest?  Q8: Was follow up complete and if not, were differences between groups in terms of their follow up adequately described and analyzed?  Q9: Were participants analyzed in the groups to which they were randomized?  Q10: Were outcomes measured in the same way for treatment groups?  Q11: Were outcomes measured in a reliable way?  Q12: Was appropriate statistical analysis used?  Q13: Was the trial design appropriate, and any deviations from the standard RCT design (individual randomization, parallel groups) accounted for in the conduct and analysis of the trial? |

| Table S6 Characteristics of included studies | | | | | | | | | | | |
| --- | --- | --- | --- | --- | --- | --- | --- | --- | --- | --- | --- |
| Author (Country code) | Design | Inclusion  period | Biopsies in the study (n) | Biopsies relevant for this review (n) | Mean age | Female ^(%)^ | Mean blood pressure (mmHg)* | Mean Cr  (mg/dl)* | Mean Hgb (g/dL)* | Biopsy performed by | Needle type |
| Studies with usage of both 16G and 18G needles. | | | | | | | | | | | |
| Arora et al. (IN) ^30^ | RCT | 2007-2008 | 50 | 16G: 25  18G: 25 | NA  NA | NA  NA | NA  NA | NA  NA | NA  NA | NA  NA | NA  NA |
| Lubomirova et al (BG) ^31^ | R Cohort | 2009-2013 | 230 | 16G: 170  18G: 60 | 45.5**  45.5** | 48.3**  48.3** | <150/90  <150/90 | NA  NA | NA  NA | NA  NA | Automated  Automated |
| Kitterer et al. (DE) ^32^ | R Cohort | 2008-2012 | 205 | 16G: 54  18G: 117 | 58**  58** | 39.0**  39.0** | 130/80  130/80 | 2.1**  2.1** | 11.6**  11.6** | Nephro.  Nephro. | Automated  Automated |
| Mai et al. (AU) ^33^ | R Cohort | 2001-2010 | 934 | 16G: 753  18G: 181 | 51.0  53.0 | 47.0  45.0 | NA  NA | 1.70  1.47 | NA  NA | Nephro.  Radio. | Automated  Automated |
| Altindal et al. (TR) ^34^ | R Cohort | 2000-2013 | 290 | 16G: 25  18G: 265 | 39.9**  39.9** | 39.3**  39.3** | NA  NA | NA  NA | NA  NA | NA  NA | Automated  Automated |
| Giniyani et al. (US) ^35^ | R Cohort | 2015-2023 | 137 | 16G: 50  18G: 87 | 44.0  48.0 | NA  NA | <140/90  <140/90 | NA  NA | NA  NA | Nephro.  Nephro. | NA  NA |
| Studies with 16G needles | | | | | | | | | | | |
| Brardi et al. (IT) ^36^ | R Cohort | 2012-2017 | 50 | 50 | 52.7 | 36.0 | NA | 1.66 | 12.40 | NA | Automated |
| Chancharoenthana et al. (TH)^37^ | RCT | 2014*** | 30 | 30 | 53.6 | 60.0 | 150/90 | 3.35 | 10.80 | Nephro. | Automated |
| Chunduri et al. (US) ^10^ | P Cohort | 2010-2013 | 137 | 55 | 48.0 | 65.0 | 134/78 | 2.10 | 11.40 | Nephro. | Automated |
| Shidham et al. (US) ^38^ | R Cohort | 1981-2001 | 645 | 645 | 42.0 | 50.0 | 141/85 | 3.00 | NA | Nephro. | Mixed manual and automated |
| Bataille et al. (FR) ^39^ | R Cohort | 2006-2010 | 943 | 136 | 52.2 | 36.0 | <140/90 | NA | NA | Nephro. | Automated |
| Lim et al. (SG) ^40^ | R Cohort | 2011-2015 | 184 | 184 | 54.1 | 44.0 | 139/73 | 3.21 | 10.60 | NA | Automated |
| Wang et al. (CN) ^41^ | R+P Cohort | 2010-2012 | 1 985 | 1 563 | 40.0 | 39.5 | 132/83 | 1.35 | 12.70 | NA | Automated |
| Beniwal et al. (IN) ^42^ | R Cohort | 2012-2017 | 230 | 230 | 64.0 | 29.6 | NA | NA | NA | Nephro. | Automated |
| MacKinnon et al. (GB-SCT) ^43^ | R Cohort | 2000-2007 | 1 120 | 1 120 | 56.0 | 40.1 | NA | 1.33 | 11.75 | Nephro. | Automated |
| Takeuchi et al. (JP) ^44^ | R Cohort | 2013-2017 | 456 | 456 | 65.7 | 37.7 | 136/76 | 1.46 | 12.40 | Nephro. | Automated |
| Al-Hweish et al. (SA) ^45^ | RCT | 2004-2006 | 44 | 44 | NA | 48.0 | NA | NA | NA | Nephro. | Automated |
| Munib et al. (PK) ^46^ | R Cohort | 2013-2015 | 120 | 120 | 28.2 | 31.6 | NA | NA | NA | Nephro. | Automated |
| Munoz et al. (MX) ^47^ | R Cohort | 1998-2008 | 623 | 623 | 34.4 | 70.5 | NA | 1.70 | 12.20 | Nephro. | Automated |
| Sousanieh et al. (US) ^48^ | P Cohort | 2002-2019 | 592 | 255 | 46.0 | 42.0 | 134/80 | 2.20 | 11.40 | Nephro. | Automated |
| Gesualdo et al. (IT) ^49^ | RCT+Cohort | 2005-2007 | 110 | 110 | 45.7 | NA | 130/83 | NA | 12.86 | Nephro. | Automated |
| Constantin et al. (CA) ^11^ | R Cohort | 2005-2007 | 121 | 66 | 54.6 | 50.0 | NA | 2.09 | 12.2 | NA | Automated |
| Xu et al. (CN) ^50^ | P Cohort | 2016-2017 | 218 | 218 | 45.7 | 52.3 | <160/90 | 0.92 | 12.54 | Radio. | Automated |
| Pendon-Ruiz de Mier et al. (ES) ^51^ | P Cohort | 2009-2013 | 241 | 241 | 49.0 | 44.0 | NA | 2.90 | 12.20 | Nephro. | Semi-automated |
| Chikamatsu et al. (J P) ^52^ | R Cohort | 2013-2016 | 252 | 252 | 62.0 | 39.0 | 139/78 | NA | 12.30 | Nephro. | Automated |
| Lees et al. (GB-SCT) ^53^ | R Cohort | 2000-2014 | 2 563 | 2 563 | 57.0 | 42.6 | 139/77 | NA | 11.60 | Nephro. | Automated |
| Fontana et al. (IT) ^54^ | R Cohort | 2010-2020 | 750 | 750 | 52.2 | 41.2 | 134/77 | 1.64 | 11.70 | Nephro. | Automated |
| Granata et al. (IT) ^55^ | R Cohort | 1995-2009 | 561 | 561 | 45.9 | 43.0 | NA | NA | NA | Nephro. | Automated |
| Torigoe et al. (JP) ^56^ | R Cohort | 2017-2020 | 238 | 238 | 54.0 | 46.6 | 128/77 | NA | 12.20 | Nephro. | Automated |
| Mejia-Vilet et al. (MX) ^57^ | R+P Cohort | 2008-2016 | 1 205 | 1 205 | 33.0 | 68.8 | MAP 93 | 1.23 | 12.17 | NA | Manual |
| Hwang et al. (KR) ^58^ | R Cohort | 2014-2015 | 41 | 41 | 40.7 | 36.6 | NA | 2.00 | NA | Radio. | Automated |
| Chan et al. (CA) ^59^ | P Cohort | 1998-1999 | 25 | 25 | NA | NA | NA | NA | NA | Radio. | Spring loaded |
| Ori et al. (IL) ^60^ | P Cohort | 1995 | 94 | 85 | 52.9 | 47.1 | NA | 2.30 | NA | Both | Automated |
| Deniz et al. (TR) ^61^ | P Cohort | 2020-2022 | 71 | 71 | 47.9 | 53.5 | NA | 1.00 | 11.0 | Radio. | Automated |
| Jaturapisanukul et al. (TH) ^62^ | RCT | 2017-2019 | 107 | 107 | 41.6 | 66.0 | NA | 1.12 | NA | Nephro. | Automated |
| Yazici et al. (TR) ^63^ | R Cohort | 2001-2022 | 19 | 19 | 27.4 | 100 | NA | 0.61 | NA | Nephro. | NA |
| Goto et al. (JP) ^64^ | R Cohort | 2018-2023 | 213 | 213 | 56.0 | 48.4 | MAP 99 | 1.06 | 12.7 | Nephro. | Automated |
| Sethi et al. (IN) ^65^ | RCT | 2021-2022 | 80 | 80 | 44.1 | 37.5 | NA | 4.42 | 10.12 | Neprho. | Automated |
| Studies with 18G needles | | | | | | | | | | | |
| Kim et al. (KR) ^66^ | RCT | 1994-1997 | 166 | 99 | 35.7 | 46.5 | 126/80 | NA | 12.10 | Nephro. | Automated |
| Jandaghi et al. (IR) ^67^ | RCT | 2015-2016 | 166 | 166 | 43.0 | 46.4 | NA | NA | NA | Radio. | Semi-automated |
| Soares et al. (US) ^68^ | R Cohort | 1996-2006 | 289 | 284 | 57.4 | 43.6 | NA | 1.73 | 11.87 | Radio. | NA |
| Morabe-Dy et al. (PH) ^69^ | R Cohort | 2012-2015 | 984 | 984 | 38.3 | 56.2 | 118/78 | 1.60 | 12.43 | Nephro. | Automated |
| Jose et al. (IN) ^70^ | R Cohort | 2014-2018 | 432 | 432 | 39.1 | 36.3 | 134/84 | 6.00 | 9.41 | Nephro. | Automated |
| Sattari et al. (IR) ^71^ | RCT | 2017-2020 | 120 | 120 | 45.3 | 51.7 | 126/79 | 1.75 | 11.21 | Radio. | NA |
| Schorr et al. (CA) ^72^ | R Cohort | 2012-2017 | 617 | 247 | 57.0* | 36.5* | NA | NA |  | Nephro. | Automated |
| Ishikawa et al. (JP) ^73^ | R Cohort | 2001-2006 | 411 | 411 | 45.2 | 44.5 | NA | NA | 12.50 | NA | Automated |
| Mendelssohn et al. (Ca) ^74^ | P Cohort | 1992-1994 | 544 | 167 | NA | NA | NA | NA | 11.92 | Nephro. | Automated |
| Roccatello et al. (IT) ^75^ | P Cohort | 2000-2016 | 462 | 462 | 54.7 | 39.0 | NA | 1.67 | NA | Nephro. | Automated |
| Pokhrel et al. (NP) ^76^ | RCT | 2016 | 76 | 38 | 33.9 | 37.0 | 130/84 | NA | 12.10 | Nephro. | NA |
| Maya et al. (US) ^77^ | R Cohort | 2004-2005 | 129 | 65 | 43.0 | 61.0 | 137/NA | 2.60 | 11.30 | Nephro. | Automated |
| Cildag et al. (TR) ^78^ | R Cohort | 2017-2018 | 60 | 60 | 49.5 | 36.7 | <140/90 | 2.05 | 11.64 | Radio. | Automated |
| Tabatabai et al. (US) ^79^ | R Cohort | 1995-2007 | 1 116 | 1 116 | 45.1 | 56.3 | NA | 1.89 | NA | Nephro. | Semi-automated |
| Chen et al. (US) ^80^ | R Cohort | 1993-2007 | 219 | 219 | 36.5 | 85.6 | 133/80 | 1.00 | NA | Radio. | Semi-automated |
| Rollino et al. (IT) ^81^ | R Cohort | 1974-2012 | 131 | 131 | 78.7 | 45.0 | NA | 4.47 | NA | Nephro. | Automated |
| TK Chen et al. (US) ^82^ | R Cohort | 2001-2012 | 11 | 11 | 24.0 | 100 | NA | 0.60 | 9.90 | Nephro. | Semi-automated |
| Monahan et al. (US) ^83^ | R Cohort | 2005-2015 | 2 204 | 2 204 | 59.0 | 42.1 | 141/80 | 2.10 | NA | Radio. | Automated |
| Vu et al. (US) ^84^ | R Cohort | 2006-2021 | 42 | 42 | 52.9 | 45.2 | NA | NA | 9.91 | Radio. | NA |
| Al-Balas et al. (US) ^85^ | R Cohort | 2017-2019 | 147 | 147 | 48.0 | 49.0 | 134/81 | NA | 12.30 | Nephro. | Automated |
| M Schorr et al. (CA) ^86^ | R Cohort | 2018-2023 | 400 | 142 | 56.0 | 40.2 | NA | NA | NA | Nephro. | Automated |
| Komatsu et al. (JP) ^87^ | R Cohort | 2016-2022 | 224 | 143 | 64.0 | 48.3 | NA | NA | 11.4 | Nephro. | Automated |
| Ravizzini et al. (BR) ^88^ | R Cohort | 2008-2021 | 445 | 216 | 40.2 | NA | NA | NA | NA | Radio. | Semi-automated |
| Riehl et al. (DE) ^8^ | R Cohort | 1990-1993 | 458 | 134 | 45.9 | 43.0 | NA | NA | NA | NA | Automated |
| *Before the biopsy. **Data only available for the whole cohort. ***Study period not mentioned, and the presented year is time of publication.  Country codes: AU, Australia. BG, Bulgaria. BR, Brazil. CA, Canada. CN, China. DE, Germany. ES, Spain. FR, France. GB-SCT, Scotland. IL, Israel. IN, India. IR, Iran. IT, Italy. JP, Japan. KR, South Korea. MX, Mexico. NP, Nepal. PH, Philippines. PK, Pakistan. SA, Saudi Arabia. SG, Singapore. TH, Thailand. TR, Turkey. US, United States.  Cr, Creatinine. G, Gauge. Hgb, hemoglobin. NA, Not Applicable. Nephro., Dept. of Nephrology. P, prospective. R, retrospective. Radio., Dept. of Radiology. RCT, Randomized controlled trial. | | | | | | | | | | | |

| Figure S3 Proportion of major complications stratified by needle size for studies with high quality |
| --- |
| 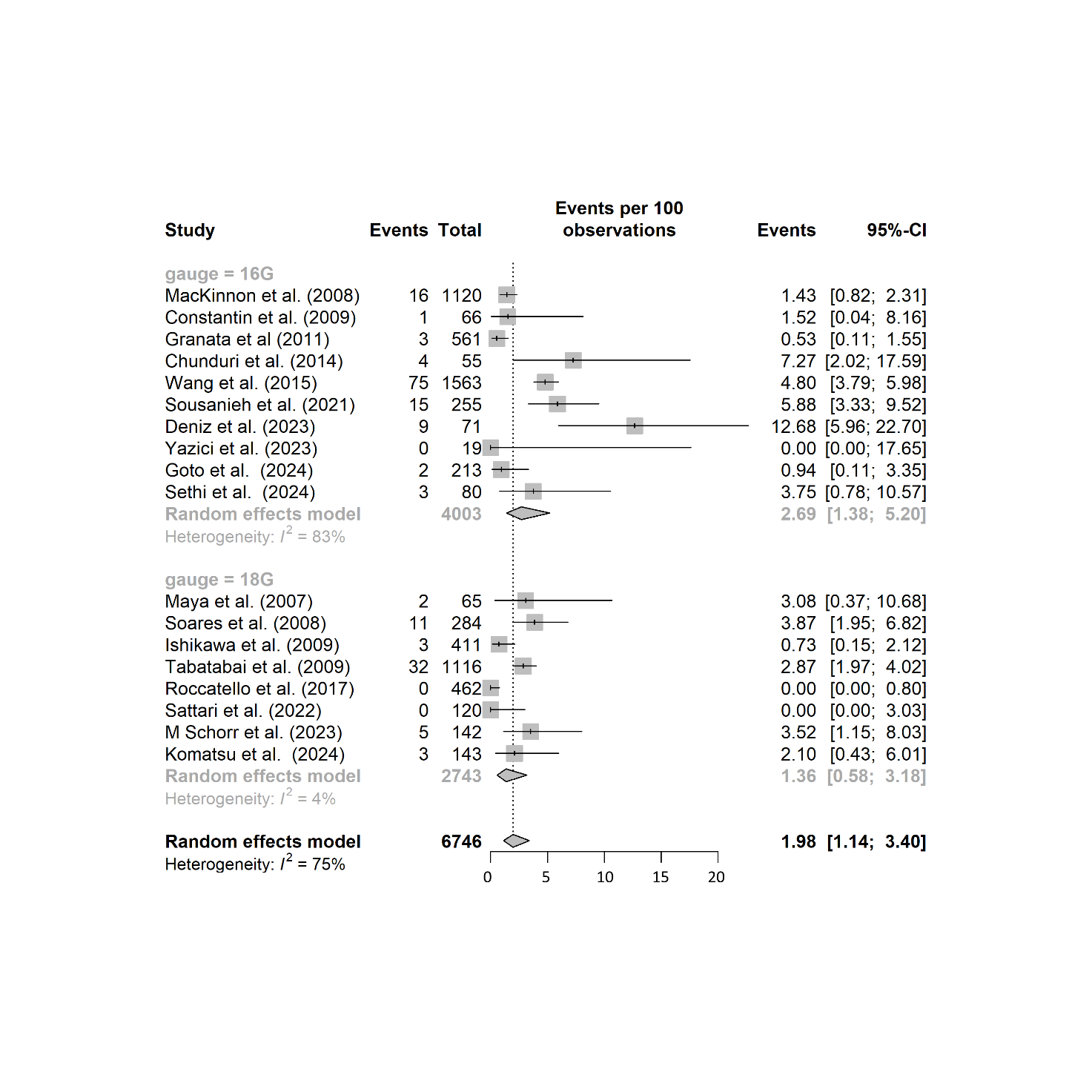 |
| Random effects model testing difference between the two needle sizes and Major bleeding complications.  CI, Confidence Interval. I², Heterogeneity. |

| Figure S4 Risk ratio of major complications stratified by needle size |
| --- |
| 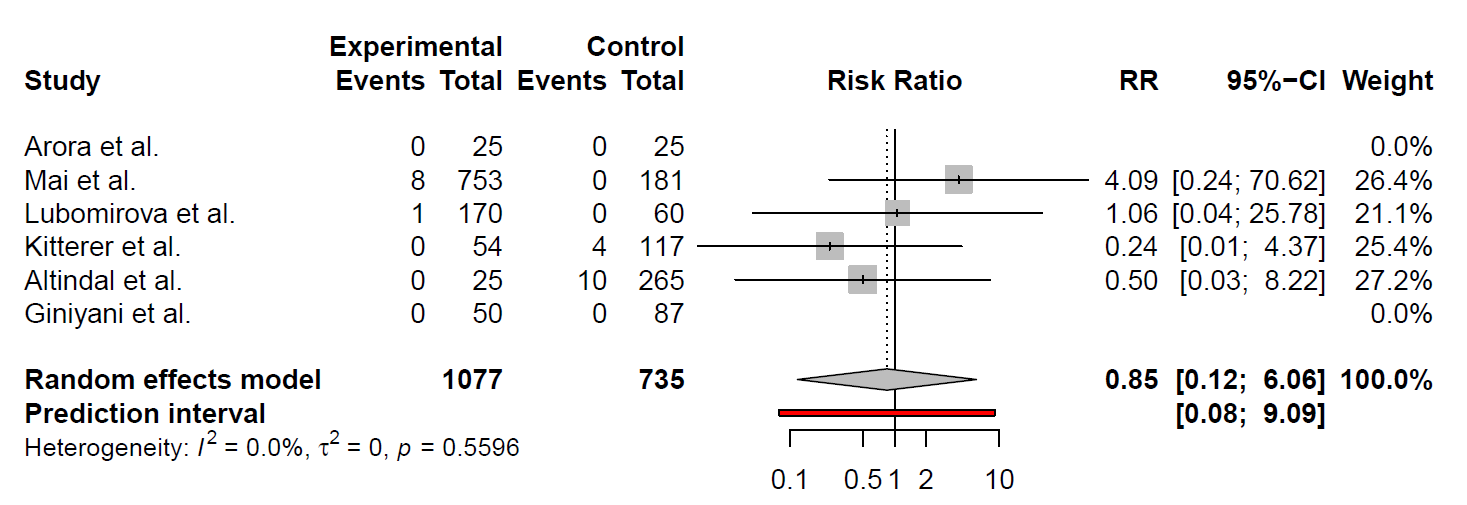 |
| Experimental = biopsies performed with 16G. Control = biopsies performed with 18G. RR, risk ratio. |

| Figure S5 Proportion of transfusion by needle size |
| --- |
| 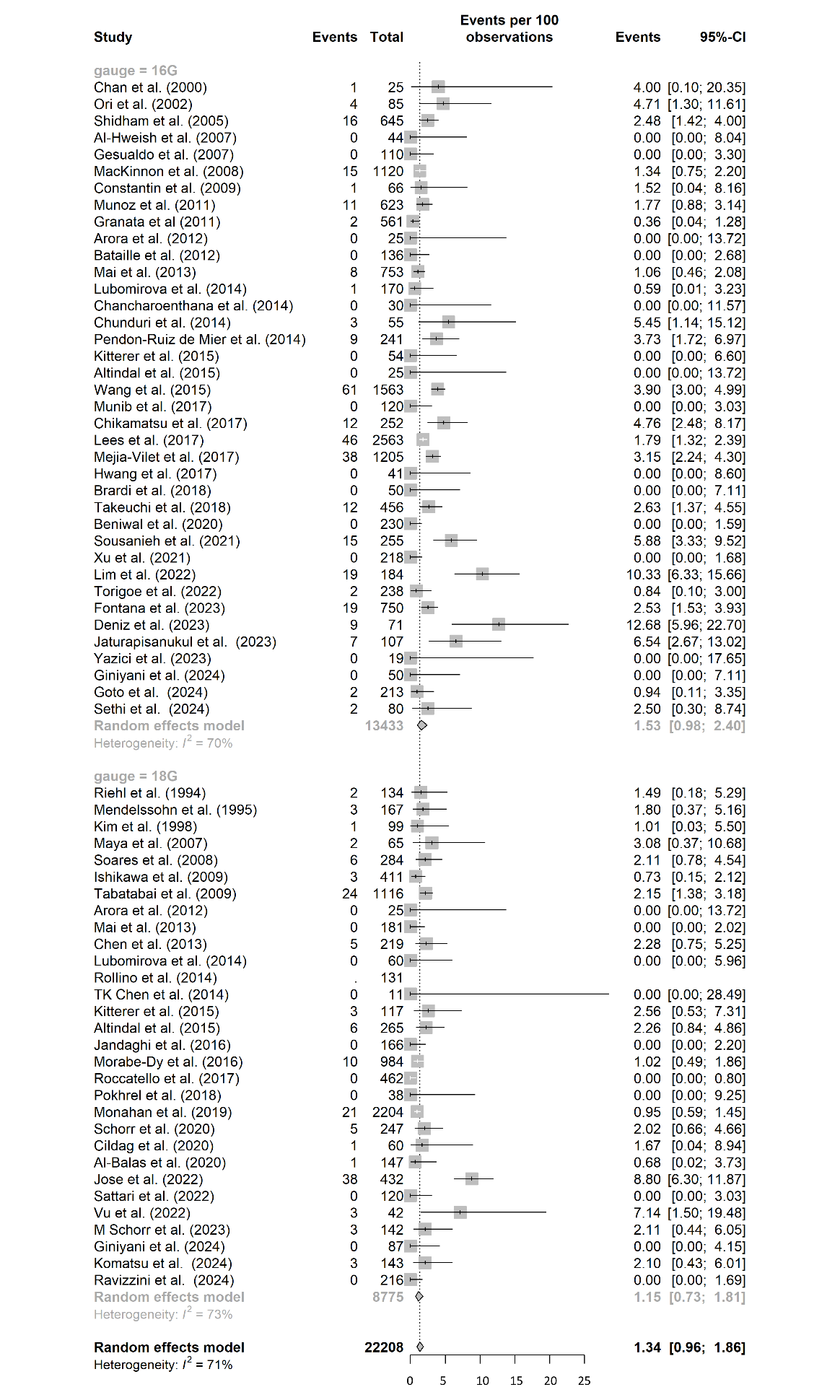 |
| Random effect model testing difference between the two needle sizes and transfusion |

| Figure S6 Proportion of embolization by needle size |
| --- |
| 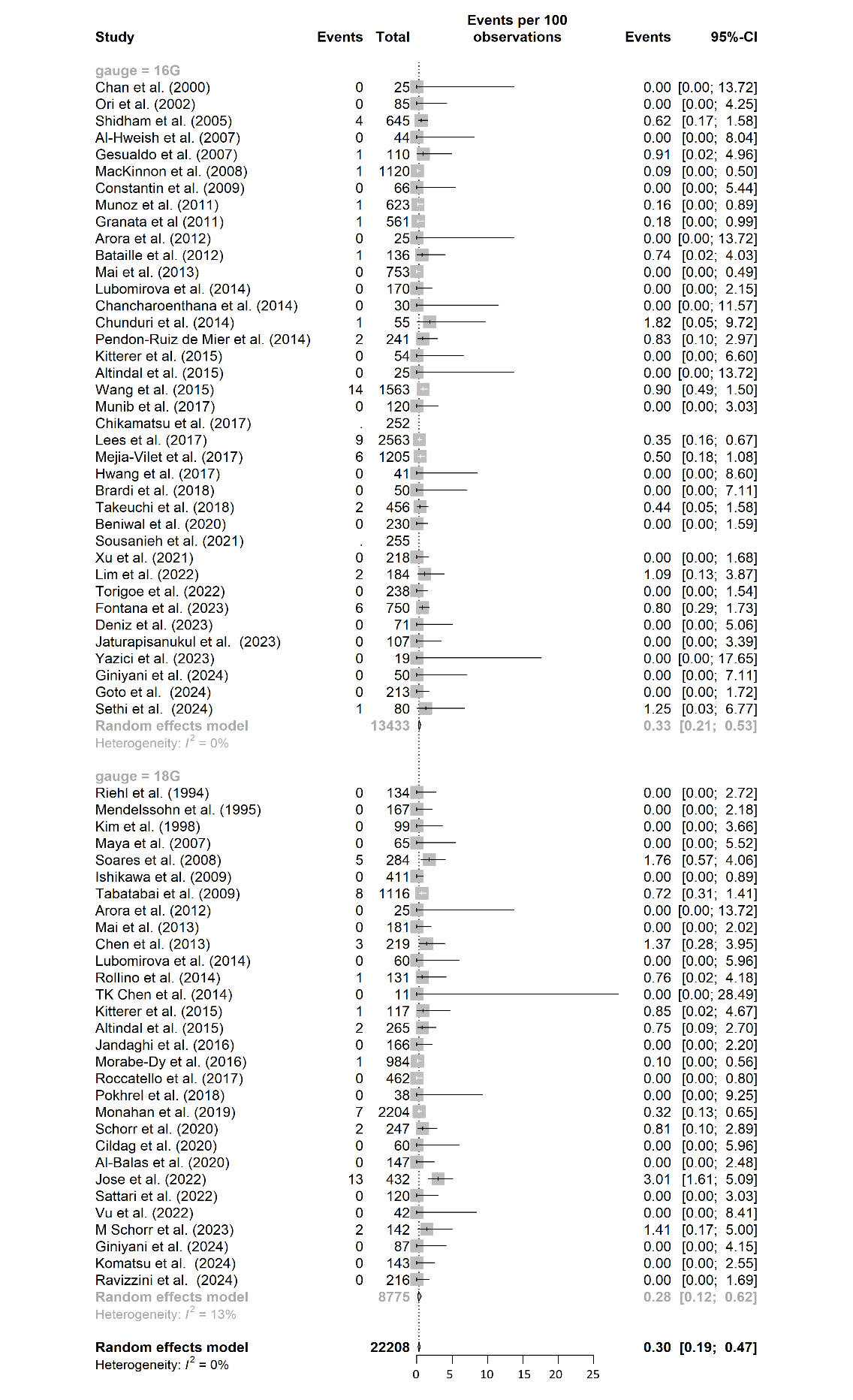 |
| Random effect model testing difference between the two needle sizes and embolization |
| Figure S7 Proportion of death stratified by needle size |
| 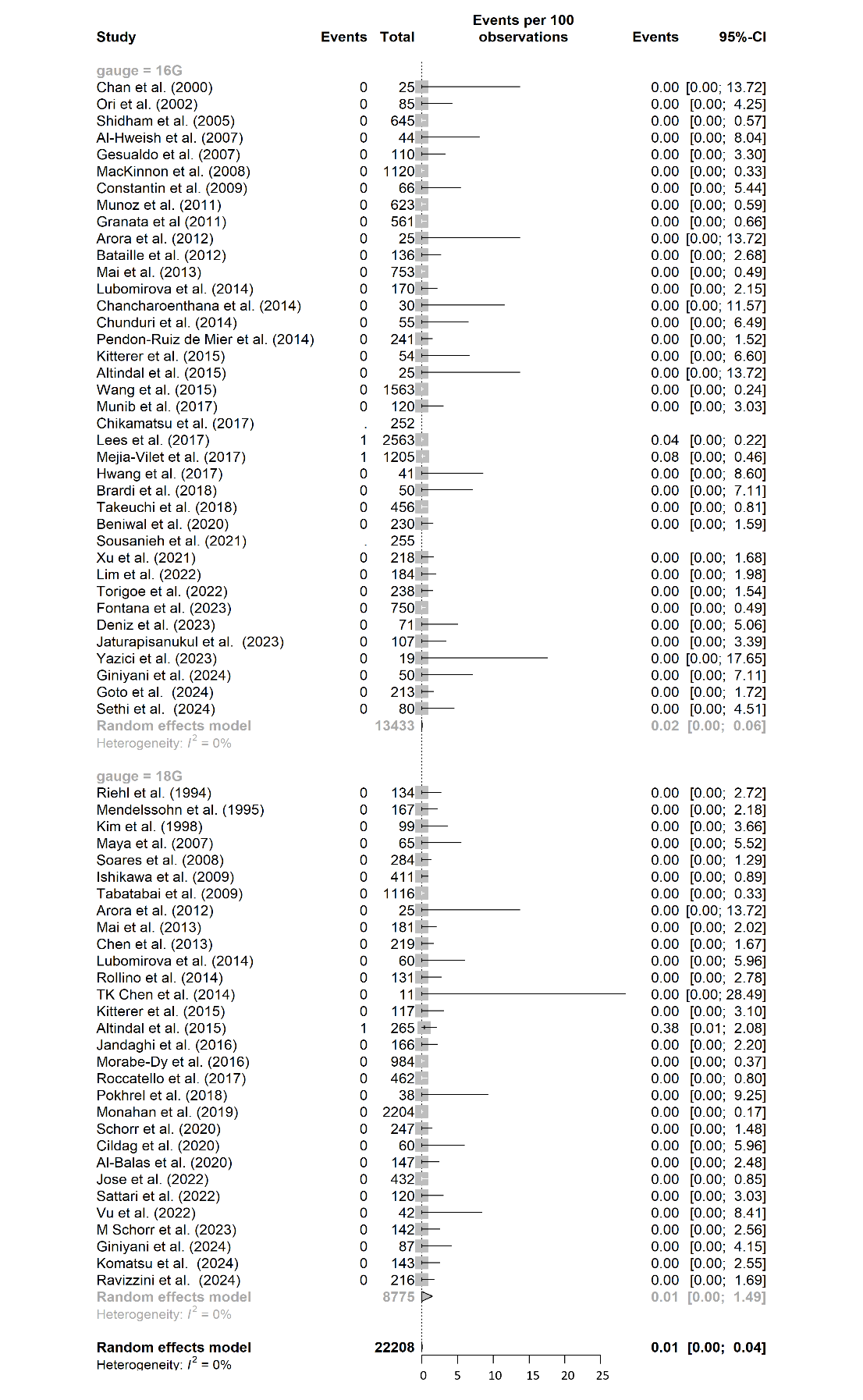 |
| Random effect model testing difference between the two needle sizes and death |

| Figure S8 Proportion of macroscopic haematuria stratified by needle size |
| --- |
| 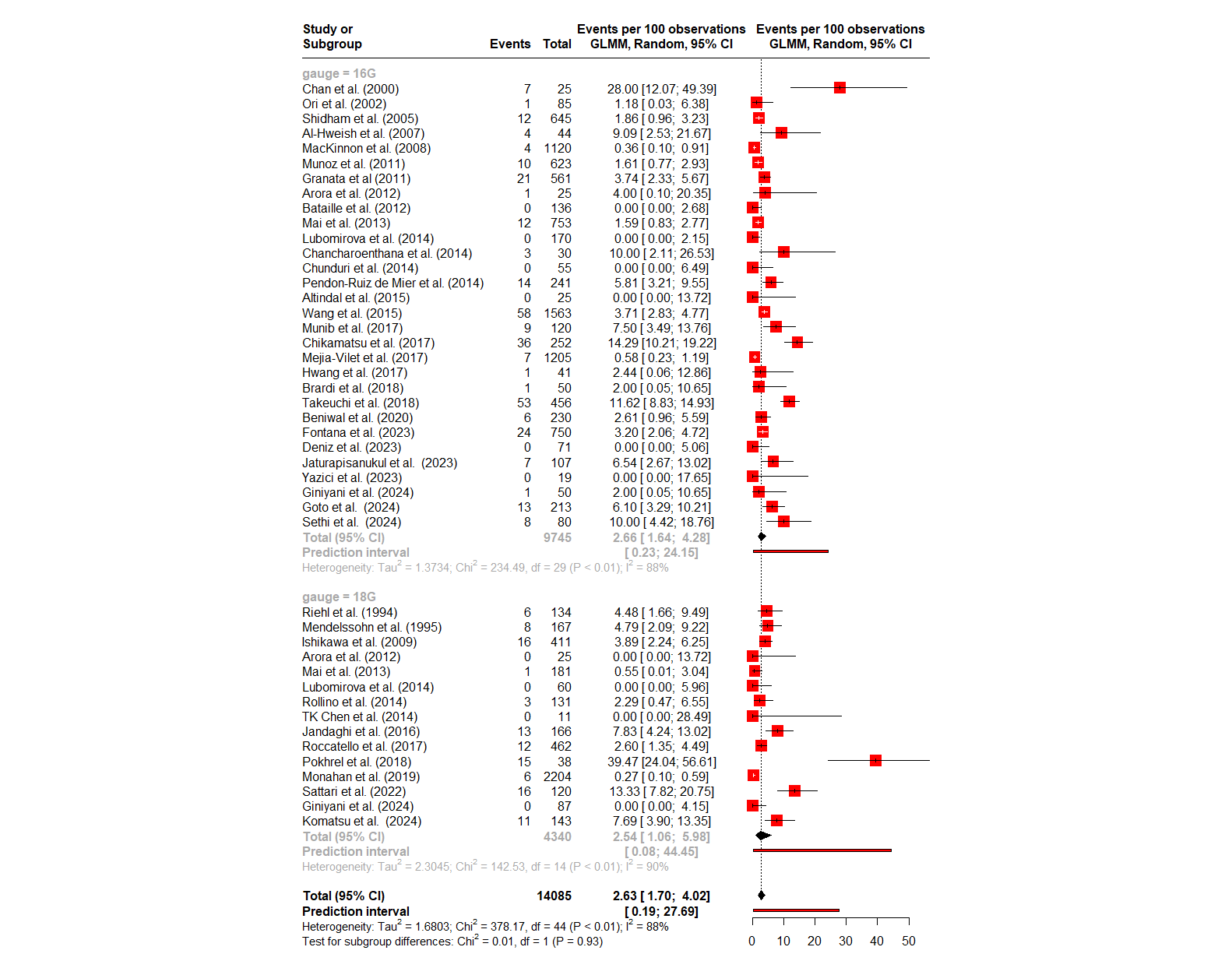 |
| Random effect model testing difference between the two needle sizes and macroscopic haematuria |

| Figure S9 Proportion of hematomas stratified by needle size |
| --- |
| 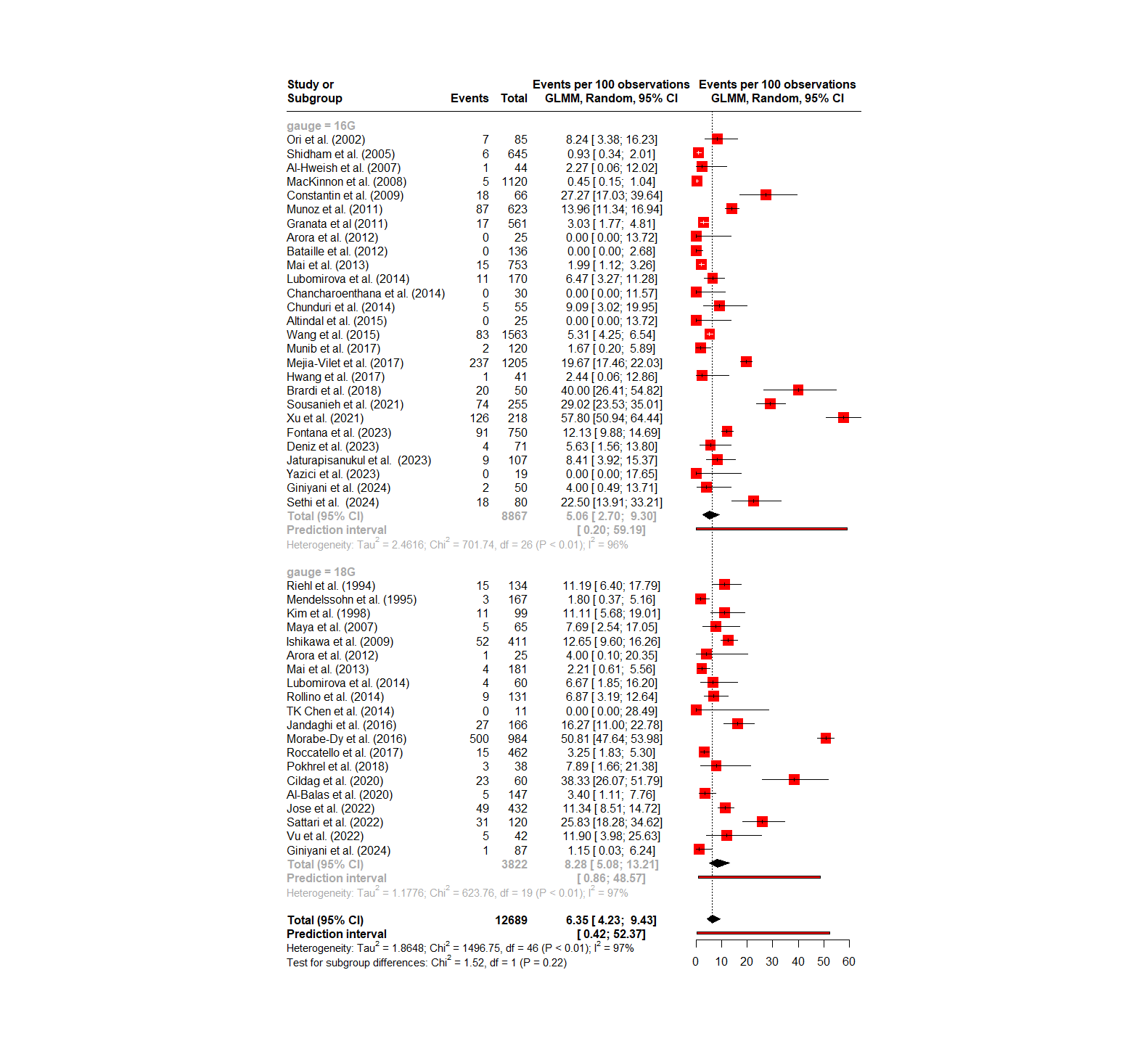 |
| Random effect model testing difference between the two needle sizes and the haematomas |

| Figure S10 Proportion of total complications (haematomas, macroscopic haematuria, transfusion, embolization, nephrectomy, other surgery, and/or death) stratified by gauge size | | | | | |
| --- | --- | --- | --- | --- | --- |
| 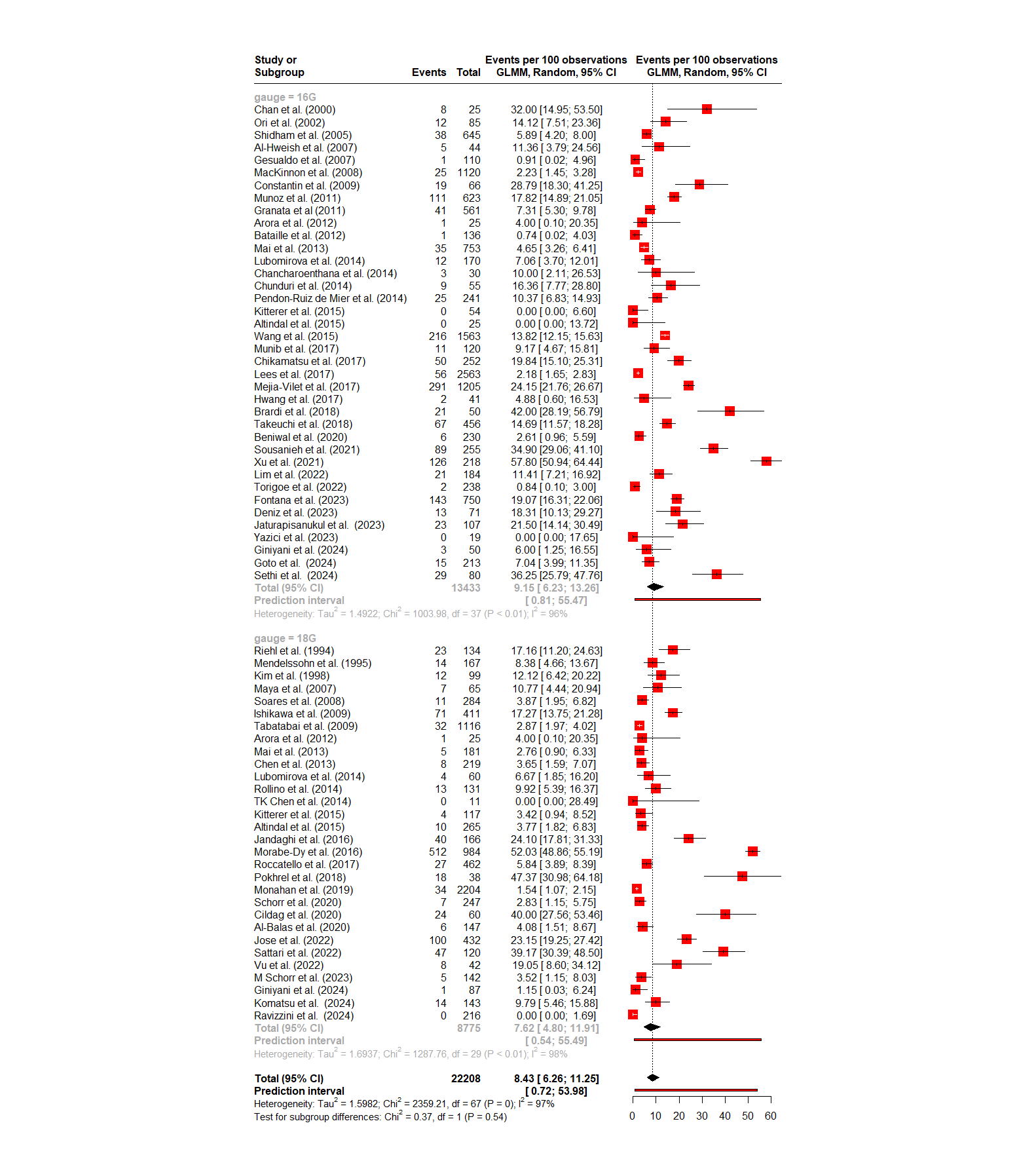 | | | | | |
| Random effect model testing difference between the two needle sizes and one or more of all registered complications. | | | | | |
| Table S7 Univariable meta-regression analysis for major complications | | | | |  |
| ***Variable*** | ***K*** | ***Coefficient (95% CI)*** | | ***P-value*** |  |
| Age | 62 | 0.0014 | (-0.03; 0.04) | 0.94 |  |
| Sex, female (%) | 59 | 0.011 | (-0.01; 0.04) | 0.38 |  |
| AKI | 18 | 0.042 | (0.002; 0.08) | **0.040** |  |
| Nephrotic | 21 | -0.032 | (-0.08; 0.01) | 0.15 |  |
| Systolic BP | 23 | 0.063 | (-0.002; 0.13) | 0.057 |  |
| Diastolic BP | 22 | -0.03 | (-0.65; 0.10) | 0.65 |  |
| Creatinine | 40 | 0.30 | (-0.006; 0.61) | **0.055** |  |
| eGFR | 20 | -0.02 | (-0.05; -0.002) | **0.035** |  |
| Hgb | 41 | -0.63 | (-0.94; -0.32) | **<0.001** |  |
| Number of biopsies | 68 | 0.0003 | (-0.0003; 0.0009) | 0.23 |  |
| Number of passes | 39 | -0.03 | (-0.67; 0.60) | 0.92 |  |
| Dept. performing the biopsy: | 55 |  |  |  |  |
| - Nephrology - Radiology - Mixed |  | -4.12  -0.26  1.02 | Ref.  (-1.02; 0.55)  (-1.28; 3.32) | Ref.  0.53  0.39 |  |
| Needle type:   - Automated - Semiautomated - Manual | 59 | -4.11  -0.12  0.27 | Ref.  (-1.2; 0.96)  (-1.52; 2.05) | Ref.  0.83  0.77 |  |
| AKI, Acute kidney injury. BP, blood pressure. eGFR, estimated glomerular filtration rate. Hgb, haemoglobin. K, number of study arms included in the analysis. Dept., department. | | | | |  |

| Table S8 Univariable meta-regression analysis for transfusion | | | | |
| --- | --- | --- | --- | --- |
| ***Variable*** | ***K*** | ***Coefficient (95% CI)*** | | ***P-value*** |
| Age | 61 | 0.007 | (-0.03; 0.04) | 0.73 |
| Sex, female (%) | 58 | 0.01 | (-0.01; 0.04) | 0.35 |
| AKI | 18 | 0.04 | (-0.001; 0.08) | 0.059 |
| Nephrotic | 21 | -0.03 | (-0.08; 0.009) | 0.12 |
| Systolic BP | 23 | 0.07 | (0.0008; 0.13) | **0.047** |
| Diastolic BP | 22 | -0.04 | (-0.18; 0.09) | 0.55 |
| Creatinine | 40 | 0.35 | (0.04; 0.66) | **0.025** |
| eGFR | 20 | -0.022 | (-0.043; -0.002) | **0.035** |
| Hgb | 41 | -0.62 | (-0.93; -0.31) | **<0.001** |
| Number of biopsies | 67 | 0.0002 | (-0.0003; 0.0008) | 0.40 |
| Number of passes | 38 | 0.04 | (-0.63; 0.70) | 0.91 |
| Dept. performing the biopsy: | 54 |  |  |  |
| - Nephrology - Radiology - Mixed |  | -4.29  -0.26  1.19 | Ref.  (-1.07; 0.56)  (-1.06; 3.43) | Ref.  0.53  0.30 |
| Needle type:   - Automated - Semiautomated - Manual | 58 | -4.26  -0.22  0.26 | Ref.  (-1.29; 0.86)  (-1.50; 2.02) | Ref.  0.69  0.77 |
| AKI, Acute kidney injury. BP, blood pressure. eGFR, estimated glomerular filtration rate. Hgb, haemoglobin. K, number of study arms included in the analysis. Dept., department. | | | | |

| Table S9 Univariable meta-regression analysis for macroscopic haematuria | | | | |
| --- | --- | --- | --- | --- |
| ***Variable*** | ***K*** | ***Coefficient (95% CI)*** | | ***P-value*** |
| Age | 39 | 0.002 | (-0.04; 0.05) | 0.93 |
| Sex, female (%) | 38 | -0.03 | (-0.07; 0.009) | 0.13 |
| AKI (%) | 13 | -0.02 | (-0.08; 0.03) | 0.40 |
| Nephrotic (%) | 17 | 0.006 | (-0.03; 0.05) | 0.76 |
| Systolic BP | 11 | -0.11 | (-0.28; 0.06) | 0.22 |
| Diastolic BP | 11 | 0.04 | (-0.30; 0.38) | 0.83 |
| Creatinine | 27 | 0.36 | (-0.16; 0.87) | 0.17 |
| eGFR | 12 | -0.01 | (-0.05; 0.03) | 0.52 |
| Hgb | 23 | -0.03 | (-0.83; 0.78) | 0.94 |
| Number of biopsies | 45 | -0.001 | (-0.002; -0.0002) | **0.02** |
| Number of passes | 27 | 0.45 | (-0.26; 1.16) | 0.22 |
| Dept. performing the biopsy: | 35 |  |  |  |
| - Nephrology - Radiology - Mixed |  | -3.36  -0.23  -1.38 | Ref.  (-1.43; 0.97)  (-4.73; 1.98) | Ref.  0.70  0.42 |
| Needle type:   - Automated - Semiautomated - Manual | 38 | -3.65  0.63  0.84 | Ref.  (-1.11; 2.38)  (-1.07; 2.75) | Ref.  0.48  0.39 |
| AKI, Acute kidney injury. BP, blood pressure. eGFR, estimated glomerular filtration rate. Hgb, haemoglobin. K, number of study arms included in the analysis. Dept., department. | | | | |
